# The Interphase Gap Effect in Cochlear Implant Users: Biological Basis, Parameter Selection, Analytical Methods, and Quantitative Scales

**DOI:** 10.1007/s10162-026-01041-3

**Published:** 2026-03-16

**Authors:** Shuman He, Zi Gao, Jacob J. Oleson, Ian C. Bruce

**Affiliations:** 1 Eye and Ear Institute, Department of Otolaryngology–Head and Neck Surgery, The Ohio State University, 915 Olentangy River Road, Suite 4000, Columbus, OH 43212 USA; 2 Department of Biostatistics, the University of Iowa, Iowa City, IA 52242 USA; 3 Department of Electrical and Computer Engineering, McMaster University, Hamilton, ON L8S 4K1 Canada

**Keywords:** Cochlear nerve survival, Cochlear nerve health, Interphase gap effect, Phase locking value, Analysis method, Quantitative scale

## Abstract

**Purpose:**

This study aimed (1) to determine the biological basis of the interphase gap (IPG) effect and (2) to identify the most informative parameters, analytical methods, and quantitative scales for evaluating the IPG effect in human cochlear implant (CI) users.

**Methods:**

The IPG effect was quantified using multiple parameters, analytical methods, and quantitative scales (three combinations using linear or logarithmic scales for the input and output variables) across three pediatric CI groups with differing cochlear nerve (CN) anatomy: children with cochlear nerve deficiency (CND), GJB2 mutations, and idiopathic sensorineural hearing loss (SNHL). All participants in the GJB2 and idiopathic SNHL groups had normal-sized CNs (NSCNs) in the test ear. Neural synchrony, a property depending on neural health, was assessed using phase locking value (PLV) and compared between children with CND and those with GJB2 mutations.

**Results:**

The PLV did not differ significantly between the CND and GJB2 groups, nor did it correlate with the IPG effect in GJB2 cases, regardless of parameter, analytical method, or quantitative scale. In contrast, consistent group differences in IPG effects on stimulation level offset and maximum slope of the eCAP input/output function were observed across all analytical methods and quantitative scales. The sensitivity of other eCAP measures—threshold, maximum amplitude, and overall linear slope—to group differences varied by quantitative scale.

**Conclusions:**

The IPG effect primarily reflects the number of active CN fibers rather than their health. Stimulation level offset and the IPG effect on maximum slope are robust indicators of CN fiber counts in CI users and are unaffected by the choice of quantitative scale.

**Supplementary Information:**

The online version contains supplementary material available at 10.1007/s10162-026-01041-3.

## Introduction

Cochlear implants (CIs) restore auditory perception in hearing impaired listeners by sending auditory information via electrical stimulation. The cochlear nerve (CN) is the first neural structure that receives and processes electrical stimulation before transmitting information to higher neural structures of the auditory system. In human listeners, a healthy Type I CN fiber, comprising 90–95% of CN fibers, consists of an unmyelinated soma, a myelinated peripheral axon extending toward the organ of Corti, and a myelinated central axon projecting into the auditory brainstem. In CI users, various amounts and degrees of deterioration in anatomical structures of CN fibers, such as degeneration of the myelin sheath of the peripheral axon, axonal dystrophy, loss of the peripheral axon, and ultimately complete loss of the entire CN fiber, have been reported among CI users as well as across cochlear regions within individual patients [1–5]. These deteriorations change many biophysical properties of affected CN fibers, such as membrane resistance, ion channel permeability, action potential initiation site, and action potential propagation velocity, which reduces their responsiveness to electrical stimulation (e.g., [6, 7]). It should be noted that damaged CN fibers with only the soma and central axon (i.e., unipolar CN fibers) can survive for decades following peripheral axon loss (e.g., [4, 8–10]) and still be activated by electrical stimulation (e.g., [11–15]). Therefore, they are counted in the number of available CN fibers in this study.

Theoretically, how well the CN faithfully encodes and processes electrical stimulation can be a bottleneck for CI clinical outcomes. This theoretical possibility is supported by recent scientific results demonstrating the importance of the CN function for CI clinical outcomes (e.g., [16–19]). As a result, identifying biomarkers for the functional status of the CN—which depends on both the number of available CN fibers and the responsiveness of these existing CN fibers to electrical stimulation—has recently regained attention and interest among clinicians and researchers in the field of cochlear implantation. Several parameters derived from the electrically evoked compound action potential (eCAP)—a near-field recorded response generated by CN fibers to electrical stimulation—have been proposed to be used to evaluate different aspects of CN’s functional status. Among these parameters, the sensitivity of the eCAP to changes in the interphase gap (IPG) of a biphasic pulse, referred to as the IPG effect, has been associated with the packing density of neuronal somas (cells/mm^2^) within Rosenthal’s canal after correcting for perikaryal size in guinea pigs (e.g., [20, 21]). In a later study, Ramekers et al. [22] reported that all deafened cochleae tested in their previous study evaluating the IPG effect [20] had fewer remaining CN fibers compared with healthy cochleae, but these remaining CN fibers were relatively healthy and showed no sign of degeneration. Therefore, these animal data support the notion that the IPG effect is primarily associated with the number of CN fibers activated by electrical stimulation. Consistent with these animal results, the IPG effect measured in children with cochlear nerve deficiency (CND), a unique patient population with fewer but otherwise healthy CN fibers, is significantly different from that measured in their matched peers with typical sensorineural hearing loss (SNHL) and normal-sized CNs [23, 24]. Collectively, these results indicate a robust relationship between the IPG effect and the number of available CN fibers in both animal models and human CI users. These experimental results are consistent with recent simulation data reported in Takanen et al. [25]. However, Brochier et al. [26] recently proposed that the IPG effect reflects CN health, which is affected by the anatomical and physiological integrity of existing CN fibers, rather than the number of existing CN fibers. In contrast, simulation results from Zhang et al. [27] demonstrated an inconsistent association between the IPG effect and peripheral axon degeneration, suggesting that the IPG effect may not be a reliable indicator of CN health. Taken together, the biological basis underlying the IPG effect remains controversial, which represents a critical knowledge gap in the field.

Regardless of its biological underpinnings, the IPG effect is indicative of CN’s functional status and associated with CI clinical outcomes (e.g., [19, 28]). Therefore, its potential as a clinical and scientific tool remains. Unfortunately, despite ongoing research, consistent evidence is still lacking regarding the most informative and scientifically robust parameters, analytical methods, or quantitative scales for evaluating the IPG effect in human CI users, which represents another critical knowledge gap. Specifically, parameters used in different studies include changes in the eCAP threshold (i.e., the lowest stimulation level to evoke an eCAP response) [23, 29, 30], the stimulation level to elicit the same eCAP amplitude (i.e., stimulation level offset) [19, 28, 31], the N1 latency [24], the slope of the eCAP input/output (I/O) function [23, 28–30], and the eCAP amplitude [23, 29]. Similarly, while the numerical differences between eCAP results measured at different IPGs have been used in some studies (e.g., [30]), the ratio of these changes or the logarithm of this ratio (i.e., dB) has been used in other studies (e.g., [23, 29, 31]). For the same set of experimental data, using different analysis methods may lead to different results and conclusions [32]. Consequently, these methodological differences make it challenging to compare results across studies.

To further complicate matters, two recent studies reported that the degree to which non-neural factors—such as impedance and electrode-to-modiolus distance—were accounted for in the analysis of eCAP parameters depended on the choice of quantitative scale (i.e., linear versus logarithmic) [25, 26]. However, there is disagreement between these two studies regarding which scale should be used. Specifically, based on formula-derivation results of a theoretical model, Brochier et al. [26] recommended the use of stimulation level offset quantified using a logarithmic scale for both input and output coordinates (i.e., log/log scale) to eliminate the effects of non-neural factors on IPG effect results. In alignment with these formula-derivation results, data collected in 15 guinea pigs by Prado-Guitierrez et al. [21] were reanalyzed and showed a significant correlation between the SGN density and the stimulation level offset—but only when analyzed on a log/log scale. In contrast, Takanen et al. [25] used a phenomenological 2-D computational model of an implanted cochlea to simulate the effect of reduced number of healthy CN fibers on eCAPs measured at different IPGs. Their simulation results demonstrated that the eCAP I/O function expressed on a log/log scale accentuated eCAP recording noise, particularly for cases with relatively small eCAP amplitudes. More importantly, their simulation results suggested that only the numerical difference in the slope of eCAP I/O function measured at different IPGs quantified on a linear input/output scale (i.e., linear/linear scale) was indicative of the number of CN fibers activated by electrical stimulation. The ratio between the slopes measured at different IPGs or the stimulation level offset quantified based on the method recommended by Brochier et al. [26] was not affected by the number of activated model CN fibers.

Taken together, inconsistent or even directly conflicting results have been reported across these studies. As a result, whether the IPG effect primarily reflects the number or the health of available CN fibers or how to best quantify the IPG effect in human CI users remains unknown. To address these critical knowledge gaps, we selected three pediatric CI patient populations with distinct anatomical characteristics of CN fibers, as suggested by histological results from human temporal bone studies, knowledge of inner ear embryogenesis and the functional role of connexin 26, and conducted two targeted experiments.

The first patient population is children with CND, which refers to a small (hypoplastic) or absent (aplastic) CN as revealed by high-resolution magnetic resonance imaging (MRI). CND occurs due to an arrested inner ear development during embryogenesis, which results in a partially underdeveloped CN [33]. The number of existing CN fibers depends on the time when the inner ear development stopped, with fewer developed CN fibers for earlier stopping time. In children with CND, the mean number of existing CN fibers, which was counted based on neuronal soma, is 5739 (range, 0–15,714), representing approximately 16.4% of the number of CN fibers expected at the corresponding age for normal hearing children (i.e., 35,500 [34]) [35–40]. It should be noted that the axons of existing CN fibers in children with CND are myelinated, with no apparent sign of degeneration [35, 36]. Furthermore, the cochlear duct development during embryogenesis follows a “base-to-apex” sequence, with the basal turn developed first [41–43]. As a result, children with CND have more CN fibers toward more basal regions of the cochlea [44]. Therefore, children with CND can be considered a human model characterized by a reduced number of otherwise healthy CN fibers whose distribution follows a unique “base-to-apex” decreasing pattern within the cochlea.

The second patient population is children with genetic mutations in gap junction beta-2 (*GJB2*). *GJB2* encodes the protein connexin 26, which is a member of the gap junction protein family expressed in the cochlea and the epidermis. Connexin 26 plays a crucial role in maintaining ion homeostasis within the cochlea by recycling potassium back to the endolymph after stimulation of sensory hair cells [45]. To date, there is no evidence suggesting the direct impact of the pathophysiological insult caused by GJB2 gene mutations on the CN. Consistent with this understanding in genetics, the histological results of a human temporal bone study showed that patients with GJB2 mutations had no noticeable neural damage in the CN [46]. Therefore, these children constitute a patient population with relatively healthy CNs.

The third patient population consists of typical pediatric CI users—children with profound sensorineural hearing loss (SNHL) and normal-sized CNs (NSCNs). For this patient population, the mean number of existing CN fibers is around 15,500 [47], and these existing CN fibers demonstrate different degrees of degeneration depending on the etiology [48, 49]. Therefore, these children represent a patient population whose number of CN fibers falls between those of children with CND and those with GJB2 mutations, but who exhibit poorer CN health than both groups.

In this study, we leverage the IPG dataset collected from 30 children with CND and 30 children with idiopathic SNHL and NSCNs in our previous study [23]. These data were reanalyzed using different parameters, analytical methods, and quantitative scales and compared with the newly collected IPG data from 18 ears of 14 children with GJB2 mutations. To enhance scientific rigor and ensure scientific reproducibility, neural synchrony in the CN between children with CND and children with GJB2 mutations was also compared. Neural discharge synchronization (i.e., neural synchrony) is a response property that is determined by the physiological properties of anatomic structures of CN fibers (i.e., neural health). Specifically, both axonal dystrophy and demyelination alter many neural properties (e.g., [50, 51]), which increases temporal jitter, spike latency, and conduction vulnerability of individual CN fibers (e.g., [50, 52, 53]). Variations in spike firing synchronization among individual neural fibers reduce the synchronized discharge across the population of neural fibers [54]. Furthermore, the SGN soma or central axon becomes the action potential initiation site to electrical stimulation when the peripheral axon completely degenerates (e.g., [11, 12, 55]). The amount of temporal dispersion or jitter included in action potentials varies with different initiate sites, leading to further reduction in discharge synchronization across CN fibers [12]. In this study, neural synchrony in the CN was quantified based on the phase locking value (PLV) using the method reported in He et al. [56]. The PLV quantifies the degree of synchrony in neural responses generated by target CN fibers across multiple trials/stimulations. It is influenced by temporal jitter in spike firing of individual CN fibers as well as discharge synchronization among all activated CN fibers across multiple stimulations.

To determine whether the IPG effect primarily reflects the number or the health of available CN fibers, the IPG results and PLVs were compared between two patient populations differing in the number of relatively healthy CN fibers: children with CND and children with GJB2 mutations. These comparisons tested the hypothesis that the IPG effect primarily reflects the number of available CN fibers responsive to electrical stimulation. Based on this hypothesis, it was expected that children with CND would exhibit comparable PLVs but differing IPG effects compared with children with GJB2 mutations.

To identify the most informative and scientifically robust parameters, analytical methods, or quantitative scales for evaluating the IPG effect in human CI users, the IPG results were compared among three patient populations: children with CND, children with GJB2 mutations, and children with NSCN. The most informative and scientifically robust parameters, analytical methods, or quantitative scales for evaluating the IPG effect would be those capable of capturing differences in the number of available CN fibers among patient populations, as well as across electrode locations within children with CND.

## Material and Methods

### General Method

All participants had congenital deafness and were implanted with a Cochlear™ Nucleus® device (Cochlear Ltd., Macquarie, NSW, Australia) with a full electrode insertion in the test ear. All participants had at least 6 months of listening experience with their CIs prior to testing. The anatomical status of the CN and the inner ear was assessed using high-resolution MRI and computed tomography (CT) of the temporal bones, following the same protocol and criteria described in our previous studies [48, 67]. The study was approved by the biomedical Institutional Review Board (IRB) of The Ohio State University (IRB study #: 2018H0344 and 2018N0005), and written informed consent was obtained from participants and/or their legal guardians prior to participation.

In children with CND, three electrodes with measurable eCAPs were selected for testing. These sites extended to the most apical electrode where a measurable eCAP could be recorded and were spaced approximately equally across the array. Based on their relative positions among electrodes with measurable eCAPs, the selected electrodes were categorized as “basal,” “middle,” and “apical.” For eCAP recordings in children with CND, the recording electrode was positioned two electrodes basal to the stimulating electrode, except for test electrodes 1 and 2, where the recording electrode was placed two electrodes apical to the stimulating electrode. For other pediatric participants, testing was conducted at three standard electrode locations: one basal electrode (electrode 3), one mid-array electrode (electrode 12), and one apical electrode (electrode 21). If an open or short circuit was detected at a designated electrode or if an eCAP could not be recorded at the maximum comfortable level for that electrode, the electrode location was adjusted accordingly. In these participants, eCAP responses were recorded two electrodes apical to the testing electrode. The exception was electrode 21, for which the response was recorded two electrodes basal to the testing electrode (i.e., electrode 19). The CI electrodes tested in each participant are listed in Table 1 and Supplemental Digit Content 1.

**Table 1:** Demographic information of the study participants newly tested in this study. *L* left ear, *R* right ear, *CI24RE (CA)* freedom contour advance electrode array, *PM* peri-modiolar, *LW* lateral-wall, *experiment(s) participated

Participant ID	Ear tested	Age at testing (years)	Internal device and electrode array	Electrode array type	Electrodes tested	Experiment(s) participated	
						Neural synchrony	IPG effect
CND01	L	3.9	CI422	LW	3, 12, 21	*	
CND02	R	4.5	CI422	LW	3, 12, 21	*	
CND03	L	15.9	24RE(CA)	PM	3, 12, 21	*	
CND04	L	5.5	CI512	PM	3, 6, 12	*	
CND05	R	7.2	CI522	LW	3, 12, 21	*	
CND06	L	2.1	CI512	PM	3, 12, 21	*	
CND06	R	1.8	CI512	PM	3, 12, 21	*	
CND07	L	6.9	CI512	PM	3, 12, 21	*	
CND08	L	1.8	CI512	PM	1, 4, 7	*	
CND09	L	9.7	24RE(CA)	PM	3, 12, 21	*	
CND10	L	6.4	24RE(CA)	PM	1, 6, 11	*	
CND11	R	8.5	24RE(CA)	PM	3, 12, 21	*	
CND12	L	7.7	24RE(CA)	PM	3, 12, 21	*	
CND12	R	7.7	24RE(CA)	PM	3, 12, 21	*	
CND13	R	11.8	24RE(CA)	PM	3, 12, 20	*	
G01	L	4.3	24RE (CA)	PM	3, 12, 21	*	
G02	R	8.7	24RE (CA)	PM	3, 12, 21	*	
G03	L	11.7	CI512	PM	3, 12, 21	*	*
G04	R	5.7	CI422	LW	3, 12, 21	*	
G05	R	7.3	24RE (CA)	PM	3, 12, 21	*	
G06	R	10.4	24RE (CA)	PM	3, 12, 21	*	
G07	R	14.2	CI512	PM	3, 12, 21	*	*
G08	R	4.6	24RE (CA)	PM	3, 12, 21	*	
G09	R	7.7	CI512	PM	3, 12, 21	*	*
G10	R	5.8	24RE (CA)	PM	3, 12, 21	*	
G11	R	8.5	24RE (CA)	PM	3, 12, 21	*	
G12	R	8.8	24RE (CA)	PM	3, 12, 21	*	*
G13	R	9.6	24RE (CA)	PM	3, 12, 21	*	
G14	R	9.1	24RE (CA)	PM	3, 12, 21	*	
G15	L	14.2	24RE (CA)	PM	3, 12, 21	*	*
G16	R	7.8	24RE (CA)	PM	3, 12, 21	*	
G17	L	17.5	24RE (CA)	PM	4, 12, 21	*	*
G17	R	17.5	24RE (CA)	PM	3, 12, 21	*	*
G18	L	16.0	24RE (CA)	PM	3, 12, 21	*	*
G18	R	16.4	24RE (CA)	PM	3, 12, 21	*	*
G19	L	8.1	CI532	PM	3, 12, 20	*	*
G19	R	8.4	CI532	PM	3, 12, 21	*	*
G20	L	14.6	24RE (CA)	PM	3, 12, 21	*	
G20	R	14.6	CI512	PM	3, 12, 21		*
G21	L	4.1	CI632	PM	3, 12, 21	*	*
G21	R	5.1	CI632	PM	3, 12, 21		*
G22	R	9.0	24RE (CA)	PM	3, 12, 21		*
G23	R	8.5	24RE (CA)	PM	3, 12, 20		*
G24	L	14.8	24RE (CA)	PM	3, 12, 21		*
G25	L	2.1	CI632	PM	6, 12, 21		*

The stimuli used to elicit the eCAP were charge-balanced, cathodic-leading, biphasic pulses. The IPG effect was measured using a pulse phase duration of 25 μs/phase in six ears of five children with GJB2 mutations. For all other tests, a pulse phase duration of 50 μs/phase was used. All eCAP results included in this study were obtained using the advanced neural response telemetry (NRT) function that is implemented in the Custom Sound EP (v. 5.1 or v. 6.2) software (Cochlear Ltd, Macquarie, NSW, Australia). Responses were recorded using a two-pulse forward-masking paradigm [80]. The stimulus was presented to individual CI electrodes in a monopolar-coupled stimulation mode via a Freedom or N5 sound processor interfaced with a programming pod. Parameters used for eCAP measures included a masker-probe interval of 400 µs, an effective sampling rate of 20,492 Hz, and sampling delays ranging between 98 and 122 µs.

### Participants

#### Neural Synchrony

To compare neural synchrony in the CN between children with CND and children with GJB2 mutations, 13 children with CND (six girls and seven boys) and 22 children with GJB2 mutations (six girls and 16 boys) were enrolled. Both ears were tested in two children with CND (CND06 and CND12) and three children with GJB2 mutations (G17–G19). Age at testing for children with CND and children with GJB2 mutations ranged between 1.8 and 15.9 years (mean, 6.8 years; SD, 3.9 years) and between 4.1 and 17.5 years (mean, 9.8 years; SD, 4.3 years), respectively.

#### IPG Effect in Children with GJB2 Mutations

Study participants included 14 children with GJB2 mutations (three girls and 11 boys), including 10 children who also participated in the neural synchrony experiment. Age at testing for these participants ranged between 2.1 and 17.5 years (mean, 11.0 years; SD, 4.8 years). Both ears were tested for four participants (G17, G18, G19, and G21). For all other participants, only one ear was tested. In addition to these newly tested participants, data collected in 30 children with CND and 30 children with NSCNs for the study reported in He et al. [23] were reanalyzed and compared with those collected from children with GJB2 mutations.

Detailed demographic information of these newly enrolled participants and electrodes tested in different experiments is listed in Table 1. Demographic information of 30 children with CND and 30 children with NSCNs whose data were reanalyzed in this study is included in Supplemental Digital Content 1. There is no overlap between the participants listed in Table 1 and Supplemental Digital Content 1.

### Procedures

#### Neural Synchrony

**Fig. 1:**
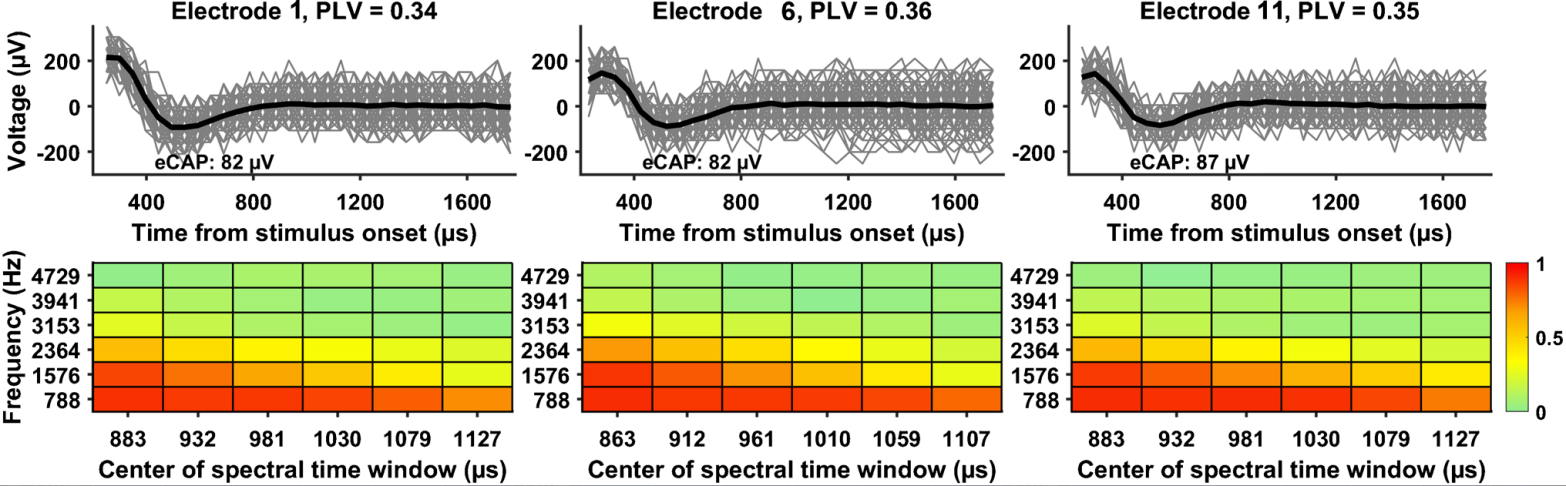
eCAP sweeps (gray) and averaged eCAPs (black) were measured at three electrode locations in one child with cochlear nerve deficiency (CND10). The tested electrode, phase locking value (PLV), and eCAP amplitude are indicated in each panel. The bottom panels display heat maps depicting PLVs as a function of frequency and center time for the six partially overlapped frames used in the Fast Fourier Transform analysis

For each participant tested in experiment 1, neural synchrony in the CN was evaluated at three CI electrode locations across the electrode array (Table 1). It was quantified using the phase locking value (PLV), a measure of trial-to-trial phase coherence among 400 eCAP sweeps evoked by a cathodic-leading, biphasic pulse with a pulse-phase duration of 50 µs presented at the maximum comfortable level (the C level). The PLV was computed using Hanning Fast Fourier Transform (FFT) functions at six frequencies ranging from 788.2 to 4729.2 Hz with a step size of 788.2 Hz, a pad-ratio of 2, and a frame size of 26 samples (1268.8 µs) using an equation in the form of

$$PLV\left(f,t\right)=\left|\frac{1}{N}\sum_{k=1}^{N}\frac{{F}_{k}(f,t)}{\left|{F}_{k}(f,t)\right|}\right|$$

where *N* denotes the number of eCAP sweeps, and ${F}_{k}(f,t)$ is the spectral estimate (i.e., complex number representing the amplitude and phase of a sinusoid obtained from the short-time Fourier transform) of trial *k* at frequency *f* for the time window *t*. The lower bound (788.2 Hz) was determined by the FFT frame duration of 1268.8 μs, which was based on the interphase latency reported for CI users with typical SNHL [57] and the 48.8-μs sampling resolution of Cochlear™ Nucleus® devices. The upper bound (4729.2 Hz) was selected based on FFT analyses of averaged eCAPs (over 400 sweeps) from adult CI users, which showed that the highest harmonic with an amplitude of at least one-quarter of the fundamental frequency occurred at 4482.6 Hz. Using the spectral resolution of 788.2 Hz determined by the frame size, this yielded an upper frequency of 4729.2 Hz for the PLV calculation. The PLV is a unitless quantity that ranges from 0 to 1 with larger numbers indicating better/stronger neural synchrony. To quantify neural synchrony in target CN fibers at each electrode location tested, a single PLV was obtained by averaging all time-frequency-specific PLVs (numbers indicated by color in rectangles of each heat map of Fig. 1) calculated at six frequencies for six partially overlapped frames that span a time window where the eCAP is expected. All details of this method have been reported previously [56].

#### IPG Effect

For children with GJB2 mutations tested in experiment 2, the eCAP I/O function was measured at each of the three selected electrodes using IPGs of 7 and 42 μs. For each study participant, the C level was measured for each testing electrode using the same procedure as reported previously [23, 44] and was used as the highest stimulation level to measure eCAPs. To obtain the eCAP I/O function, the masker pulse was presented at the C level, and the probe pulse was presented at 5–10 current levels (CLs) below the C level. The probe level was systematically decreased in steps of 1 CL for the first five steps and then in steps of 5 CLs until no eCAP response could be visually identified. The probe level was subsequently increased in steps of 1 CL until at least five consecutive stimulation levels each yielded a visually detectable eCAP with an amplitude of ≥ 5 μV.

### Data Analyses

The eCAP parameters evaluated in this study included the stimulation level offset, the maximum slope of the eCAP I/O function, the overall linear slope of the eCAP I/O function, the maximum eCAP amplitude, and the eCAP threshold. The accuracy of the N1 latency measurement is affected by the recording noise floor and sampling rate, both of which vary substantially across CI devices. Therefore, it was not evaluated in this study.

**Fig. 2:**
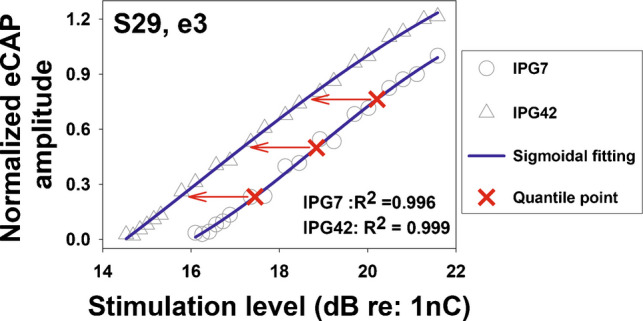
Illustration of the method used to calculate the stimulation level offset, based on example data measured at electrode 3 in one participant (S29). Different gray symbols represent normalized eCAP amplitudes measured at various stimulation levels and interphase gaps (IPGs). Black lines indicate the fitted sigmoidal functions, and the goodness-of-fit values for each IPG condition are shown in the figure. Red crosses denote the points corresponding to 25%, 50%, and 75% of the overlapping normalized eCAP amplitudes that were used to calculate the stimulation level offset

The maximum slope of the eCAP I/O function was estimated using the window method as reported in Skidmore et al. [58]. Briefly, the eCAP I/O function was first resampled to 11 points, after which eight sliding-window linear regressions were performed, each using four consecutive data points. The maximum slope across all windows was selected and used for data analyses. This maximum (steepest) slope provides a close approximation to the slope obtained by fitting a sigmoidal function to the I/O function, as both approaches estimate the first derivative of the underlying sigmoidal physiological response evaluated at its point of maximal growth (i.e., the inflection point). A key advantage of the window method is that it yields stable and interpretable slope estimates even when the I/O function is not sampled over a sufficiently wide dynamic range—particularly at high stimulation levels beyond the inflection point, which may be constrained by loudness discomfort—conditions under which sigmoidal models may fail to converge or yield poorly constrained parameter estimates. Consequently, the maximum slope exceeded the overall slope (described below), as it selectively captured the steepest region of the eCAP I/O function. The overall linear slope was estimated using linear regression of all eCAP amplitudes measured between the stimulation levels corresponding to the eCAP threshold and the C level [59]. The eCAP threshold was defined as the lowest stimulation level that elicited an eCAP response with an amplitude of 5 µV or larger [60]. The stimulus level offset was calculated using the same method as that reported in Skidmore & He [24]. Briefly, all eCAP amplitudes were normalized to the maximum eCAP amplitude at the 7 μs IPG and plotted as a function of stimulation level. The normalized eCAP I/O function was fit using a sigmoidal function [20, 44] of the form

$${\mathrm{eCAP}}_{N}={\mathrm{y}}_{0}+ \frac{\mathrm{a}}{1+{\mathrm{e}}^{-\frac{x-b}{c}}}$$

where ${\mathrm{eCAP}}_{N}$ denotes the normalized eCAP amplitude, ${\mathrm{y}}_{0}$ is the baseline normalized eCAP amplitude, *a* represents the range of normalized eCAP amplitude, *b* is the midpoint of the function, and *c* is the slope, with larger values corresponding steeper growth. The stimulation level offset was calculated as the averaged differences in stimulation level at 25%, 50%, and 75% of the overlapping eCAP amplitudes measured with IPGs of 7 μs and 42 μs, estimated based on results of sigmoidal regression. Figure 2 illustrates the method used to calculate stimulation level offset. The sigmoidal function was chosen for stimulation level offset calculation because it exhibits a stronger correlation with CN fiber counts in animal models on both linear/linear and linear/log scales compared with linear regression [58].

The maximum amplitude was quantified using linear (i.e., μV) and logarithmic (i.e., dB re: 1 µV) scaling units. The eCAP threshold was quantified using linear (i.e., nanocoulombs (nC)) and logarithmic (i.e., dB re: 1 nC) scaling units. The slopes of eCAP I/O function were quantified on three different input/output scales (i.e., μV/nC, μV/dB re: 1 nC, and dB re: 1 µV/dB re: 1 nC), and the stimulation level offset was calculated from eCAP I/O functions quantified on each input/output scale. In this study, dB_linear/log_ and dB_log/log_ denote the stimulation level offset calculated from eCAP I/O functions on a linear input/logarithmic output scale and on a logarithmic input/logarithmic output scale, respectively. The stimulation level offset calculated from eCAP I/O functions on a linear input/linear output scale was quantified in nC. In this study, each dependent variable (DV) corresponds to a specific parameter quantified on a single scale. All three scales have been used in previous studies investigating the IPG effect (e.g., [20, 25, 26, 29]). The use of these scales is supported by previous studies showing that the spike rate of single CN fibers or whole-nerve response amplitude increases approximately linearly with stimulation level when measured on a linear scale near threshold levels and on a logarithmic (dB) scale at higher stimulation levels [61, 62]. To date, there is no scientific evidence supporting the use of a linear input scale and a logarithmic output scale to quantify the eCAP I/O function. Therefore, this study did not use such scales (i.e., dB re: 1 µV/nC) to quantify the slope or to calculate the stimulation level offset.

The IPG effect was calculated by subtracting the results of these DVs measured for an IPG of 7 μs from those measured for an IPG of 42 µs (i.e., IPG effect = DV_IPG42_-DV_IPG7_). The magnitude of the IPG effect was described based on the extent of deviation from zero, with larger deviations indicating greater IPG effects.

**Fig. 3:**
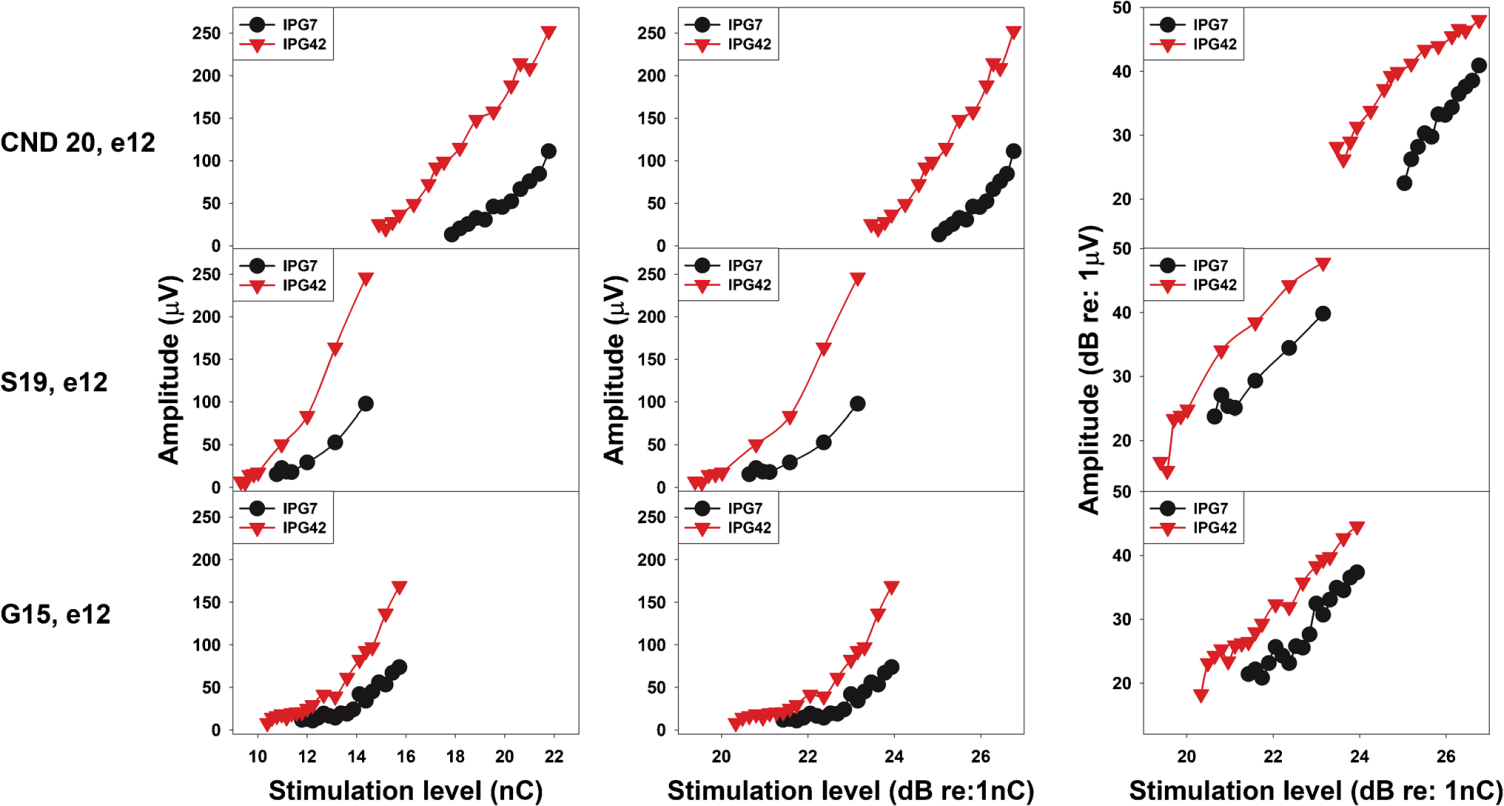
Example eCAP input–output (I/O) functions measured at interphase gaps of 7 µs (black) and 42 µs (red) at a single electrode location in three participants, where the amplitude increased roughly linearly with stimulation level across all scales. Each row shows data from one participant, with the three panels representing different input/output scales. Participant number and electrode location are indicated to the left of each row

**Fig. 4:**
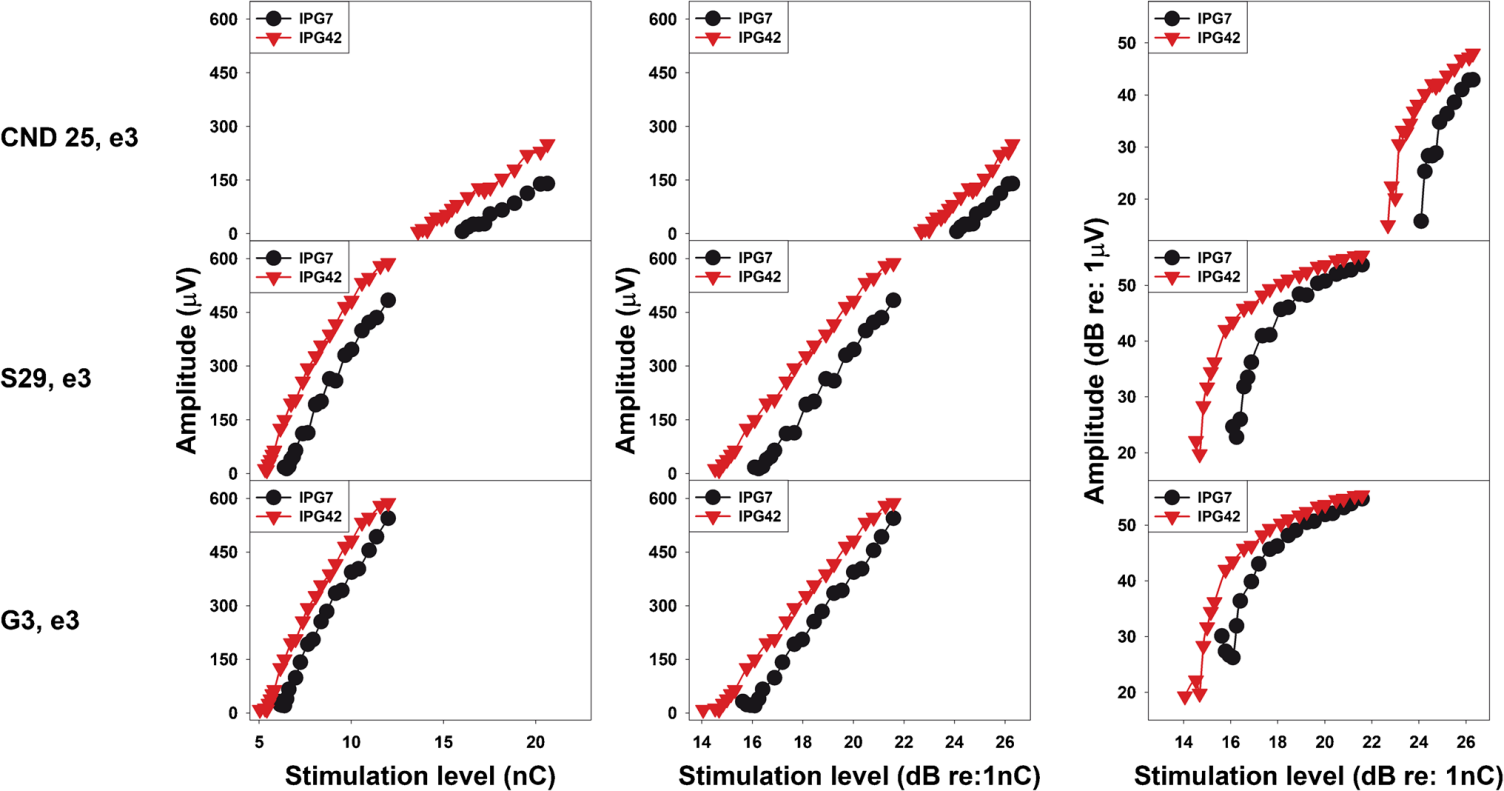
Examples of eCAP input/output (I/O) functions measured at interphase gaps of 7 µs (black) and 42 µs (red) at a single electrode location in three participants, where the eCAP I/O function exhibits saturation when plotted on a log/log scale. Each row shows data from one participant, with the three panels representing different input/output scales. Participant number and electrode location are indicated to the left of each row

Figures 3 and 4 show eCAP I/O functions measured at a single electrode in one participant from each patient group, using three different input/output scales. In Fig. 3, the eCAP I/O function shows a roughly linear increase with stimulation level for both linear and logarithmic output scales. In Fig. 4, the function remains approximately linear for the linear output scales (left and middle columns) but exhibits nonlinear saturation for the log/log scale (right column).

### Statistical Analyses

Descriptive statistics of PLVs and IPG effects on these DVs measured at different electrode locations were calculated and reported. No outliers were removed from statistical analysis for any DVs. A linear mixed effects model (LMM) was used to compare PLVs between children with CND and children with GJB2 mutations. Tukey’s honest significant difference (Tukey’s HSD) method was applied to adjust for multiple comparisons. Spearman Rank correlation tests were used to evaluate the association between the PLV and the IPG results measured in the same group of children with GJB2 mutations. Significance levels of these correlation tests were adjusted using the Bonferroni correction.

LMMs were used to compare DVs of the IPG effect measured at different electrode locations among three participant groups, with one LMM built for each DV. All LMMs included a random intercept for subject to account for repeated measures. The Benjamini-Hochberg (BH) method [63] was used to control the false discovery rate for all post hoc comparisons in experiment 2.

For all LMMs used in this study, there was a random intercept for participants, and estimations were obtained using restricted maximum likelihood with Kenward-Roger degrees of freedom. All LMMs used a correlated regression model with an unstructured correlation matrix to account for repeated observations per participant and heteroscedastic, group-specific variances to allow for unequal residual variances across groups. In addition to *t*-tests for each effect in the model, Wald Type II F-tests were performed to evaluate the overall effect of participant group on each DV. All statistical analyses for this study were performed in R software v. 4.3.0 (R Core Team, 2023, https://www.R-project.org/) using the nlme package (Pinheiro et al., 2025, https://cran.r-project.org/web/packages/nlme). Post hoc pairwise comparisons based on estimated marginal means and errors were conducted using the emmeans package (Lenth, 2023, https://cran.r-project.org/web/packages/emmeans). Statistical significance was determined at the 95% confidence level (i.e., *p* < 0.05).

## Results

### Neural Synchrony in the CN

**Fig. 5:**
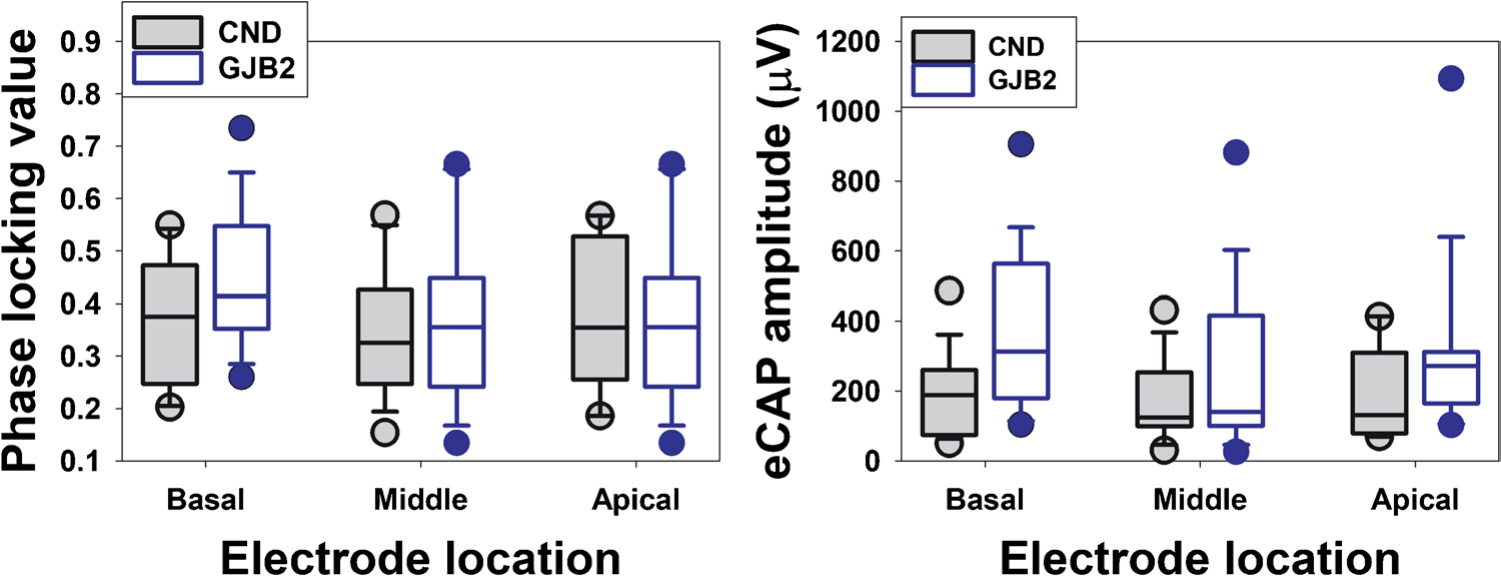
Phase locking values (PLVs) and eCAP amplitudes measured at different electrode locations in children with cochlear nerve deficiency (CND) (filled boxes) and children with biallelic gap junction beta-2 (GJB2) gene mutations (open boxes). The line inside the box and box edges represents the median and the interquartile range (IQR), spanning the 25th to 75th percentiles, respectively. Whiskers indicate the most extreme data points within 1.5 times the IQR from the lower and upper quartiles. Dots falling outside the box indicate the 5th and 95th percentiles

PLVs measured in children with CND and children with biallelic GJB2 gene mutations range from 0.15 to 0.57 (mean, 0.36; SD, 0.12) and from 0.13 to 0.75 (mean, 0.39; SD, 0.13), respectively. Amplitudes of the averaged eCAP across 400 sweeps in children with CND and children with biallelic GJB2 gene mutations range from 30.78 to 487.15 μV (mean, 181.11 µV; SD, 115.05 µV) and from 26.18 to 1158.10 µV (mean, 312.53 µV; SD, 228.67 µV), respectively. Panels (a) and (b) of Fig. 5 show the medians and inter-quartile ranges of PLVs and eCAP amplitudes measured at different electrode locations in these two patient populations, respectively.

Our previous finding in adult CI users demonstrated that larger eCAP responses were associated with higher PLV values [64]. Therefore, in the present study, PLVs were compared between children with CND and those with biallelic GJB2 gene mutations, both with and without controlling for the potential effect of response amplitude on the PLV. The results of the LMMs showed no statistically significant difference in the PLV between children with CND and children with GJB2 gene mutations, as evidenced by the nonsignificant effects of participant group (controlled for amplitude effect: ${\chi }_{(1)}^{2}$= 1.230, *p* = 0.254; not controlled for amplitude effect: ${\chi }_{(1)}^{2}$= 1.080, *p* = 0.299) on the PLV regardless of whether the significant effect of the eCAP amplitude on the PLV (${\chi }_{(1)}^{2}$= 142.270, *p* < 0.001) was controlled for. The effect of electrode location on PLV was statistically significant both when controlling for the significant effect of eCAP amplitude (${\chi }_{(2)}^{2}$= 14.575, *p* < 0.001) and when not controlling for amplitude (${\chi }_{(2)}^{2}$= 13.686, *p* = 0.001). The interaction between the effects of participant group and electrode location on the PLV did not reach statistical significance regardless of whether the significant effect of the eCAP amplitude on the PLV was controlled for (controlled for amplitude effect: ${\chi }_{(2)}^{2}$= 4.189, *p* = 0.123; not controlled for amplitude effect: ${\chi }_{(2)}^{2}$= 4.344, *p* = 0.114).

Without controlling for the effect of eCAP amplitude, post hoc analyses showed that PLVs measured at electrode 3 were significantly larger than those measured at electrode 12 (*p* = 0.015) and electrode 21 (*p* = 0.009), with no significant difference between electrodes 12 and 21 (*p* = 0.927). When the effect of eCAP amplitude was controlled for, post hoc analyses indicated that PLVs measured at electrode 3 were significantly larger than those measured at electrode 21 (*p* = 0.011). No significant differences were observed between electrodes 3 and 12 (*p* = 0.090) or between electrodes 12 and 21 (*p* = 0.586).

### Associations Between Neural Synchrony and the Interphase Gap Effect

**Fig. 6:**
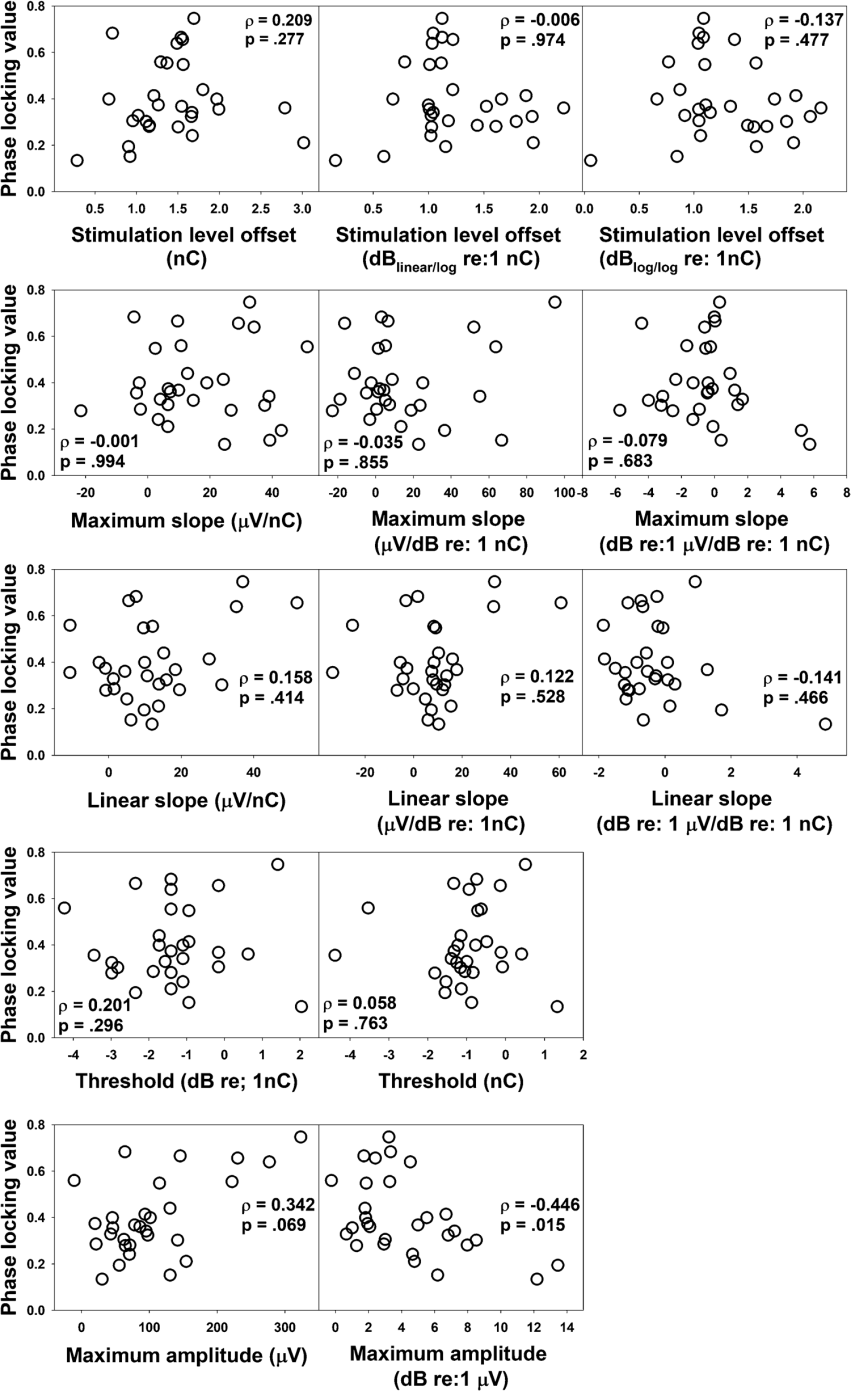
Scatter plots showing phase locking values plotted as a function of the interphase gap (IPG) effect quantified using different parameters, analytical methods, and quantitative scales in children with GJB2 mutations. The variable name under each horizontal axis refers to the IPG effect on that variable. The results of the Spearman Rank correlation test are indicated in each panel

Figure 6 shows the associations between neural synchrony in the CN, as quantified using the PLV, and the IPG effect, assessed using different parameters, analytical methods, and quantitative scales in 12 ears from 10 children with GJB2 mutations. Spearman Rank correlation tests revealed no significant association between the two metrics, regardless of how the IPG effect was quantified, after applying Bonferroni correction for multiple comparisons.

### The Interphase Gap Effect

Based on the results of LMMs after controlling the false discovery rate, all parameters calculated using different analytical methods and quantitative scales were classified into three groups based on their sensitivity to differences in the number of CN fibers both across participant groups and across electrode locations within the CND group. Details of the LMM analyses and post hoc test results are provided in Tables 2, 3, 4, and 5. For ease of reading, only summarized findings are reported in the text.

**Table 2:** Results of the linear mixed effect models (LMM) examining the relationships between interphase gap (IPG) effects measures and electrode location, patient group, and the interaction between electrode location and patient group. *IPG* interphase gap, *nC* nanocoulomb, *µV* microvolt, *dBC* decibel relative to 1 nanocoulomb, *dBV* decibel relative to 1 microvolt, *dBC*_*linear/log*_ decibel relative to 1 nanocoulomb, calculated from the input/output (*I/O*) function quantified on a linear input/logarithmic output scale, *dBC*_*log/log*_ decibel relative to 1 nanocoulomb, calculated from the I/O function quantified on a logarithmic input/logarithmic output scale. Bolded text indicates *p*-values that are considered statistically significant (*p* < 0.05).

IPG effect measure	Quantitative scale	LMM results		
		Electrode	Group	Interaction (electrode * group)
Stimulation level offset	nC	${\chi }_{(2)}^{2}$= 3.20 *p* = 0.202	${\chi }_{(2)}^{2}$= 185.44 ***p* < 0.001**	${\chi }_{(4)}^{2}$= 39.38 ***p* < 0.001**
	dBC_linear/log_	${\chi }_{(2)}^{2}$= 23.07 ***p* < 0.001**	${\chi }_{(2)}^{2}$= 41.07 ***p* < 0.001**	${\chi }_{(4)}^{2}$= 17.18 ***p* = 0.002**
	dBC_log/log_	${\chi }_{(2)}^{2}$= 25.01 ***p* < 0.001**	${\chi }_{(2)}^{2}$= 33.75 ***p* < 0.001**	${\chi }_{(4)}^{2}$= 16.01 ***p* = 0.003**
Threshold	nC	${\chi }_{\left(2\right) }^{2}$= 5.37 *p* = 0.068	${\chi }_{(2)}^{2}$= 147.00 ***p* < 0.001**	${\chi }_{(4)}^{2}$= 43.77 ***p* < 0.001**
	dBC	${\chi }_{(2)}^{2}$= 23.21 ***p* < 0.001**	${\chi }_{\left(2\right)}^{2}$=12.92 ***p* = 0.002**	${\chi }_{(4)}^{2}$= 3.55 *p* = 0.470
Max amplitude	µV	${\chi }_{(2)}^{2}$= 5.08 *p* = 0.079	${\chi }_{(2)}^{2}$= 11.04 ***p* = 0.004**	${\chi }_{(4)}^{2}$= 1.41 *p* = 0.843
	dBV	${\chi }_{(2)}^{2}$= 5.31 *p* = 0.070	${\chi }_{(2)}^{2}$= 5.22 *p* = 0.074	${\chi }_{(4)}^{2}$= 11.49 ***p* = 0.022**
Max slope	µV/nC	${\chi }_{(2)}^{2}$= 0.95 *p* = 0.621	${\chi }_{(2)}^{2}$= 27.50 ***p* < 0.001**	${\chi }_{(4)}^{2}$= 1.78 *p* = 0.777
	µV/dBC	${\chi }_{(2)}^{2}$= 1.09 *p* = 0.579	${\chi }_{(2)}^{2}$= 12.50 ***p* = 0.002**	${\chi }_{(4)}^{2}$= 0.72 *p* = 0.949
	dBV/dBC	${\chi }_{(2)}^{2}$= 1.19 *p* = 0.552	${\chi }_{(2)}^{2}$= 10.57 ***p* = 0.005**	${\chi }_{\left(4\right)}^{2}$= 8.34 *p* = 0.080
Linear slope	µV/nC	${\chi }_{(2)}^{2}$= 0.984 *p*—0.611	${\chi }_{(2)}^{2}$= 19.55 ***p* < 0.001**	${\chi }_{(4)}^{2}$= 2.29 *p* = 0.682
	µV/dBC	${\chi }_{(2)}^{2}$= 4.76 *p* = 0.093	${\chi }_{(2)}^{2}$= 0.14 *p* = 0.930	${\chi }_{(4)}^{2}$= 3.53 *p* = 0.473
	dBV/dBC	${\chi }_{(2)}^{2}$= 1.21 *p* = 0.545	${\chi }_{(2)}^{2}$= 3.74 *p* = 0.154	${\chi }_{(4)}^{2}$= 5.44 *p* = 0.245

**Table 3:** Post hoc tests for the significant main effect of patient group on interphase gap (IPG) effect measures. *IPG* interphase gap, *CND* cochlear nerve deficiency, *GJB2* gap junction beta-2 mutation, *NSCN* normal-sized cochlear nerve, *nC* nanocoulomb, *µV* microvolt, *dBC* decibel relative to 1 nanocoulomb, *dBV* decibel relative to 1 microvolt, *dBC*_*linear/log*_ decibel relative to 1 nanocoulomb, calculated from the input/output (I/O) function quantified on a linear input/logarithmic output scale, *dBC*_*log/log*_ decibel relative to 1 nanocoulomb, calculated from the I/O function quantified on a logarithmic input/logarithmic output scale, *FDR* false discovery rate. Bolded text indicates *p*-values that are considered statistically significant (*p* < 0.05).

**IPG effect measure**	**Quantitative scale**	**Patient group pair**	***t* value**	***p* value** (FDR adjusted)
Stimulation level offset	nC	CND–GJB2	*t*_(65)_ = 11.83	** < 0.001**
		CND–NSCN	*t*_(63)_ = 13.60	** < 0.001**
		GJB2–NSCN	*t*_(33)_ = 0.50	0.732
	dBC_linear/log_	CND–GJB2	*t*_(47)_ = 3.43	**0.003**
		CND–NSCN	*t*_(71)_ = 6.39	** < 0.001**
		GJB2–NSCN	*t*_(36)_ = 0.92	0.470
	dBC_log/log_	CND–GJB2	*t*_(48)_ = 3.00	**0.009**
		CND–NSCN	*t*_(70)_ = 5.81	** < 0.001**
		GJB2–NSCN	*t*_(38)_ = 1.17	0.400
Threshold	nC	CND–GJB2	*t*_(64)_ = − 9.07	** < 0.001**
		CND–NSCN	*t*_(66)_ = − 12.08	** < 0.001**
		GJB2–NSCN	*t*_(36)_ = − 0.31	0.841
	dBC	CND–GJB2	*t*_(37)_ = − 0.07	0.961
		CND–NSCN	*t*_(67)_ = − 3.52	**0.002**
		GJB2–NSCN	*t*_(35)_ = − 1.44	0.276
Max amplitude	µV	CND–GJB2	*t*_(46)_ = − 2.46	**0.033**
		CND–NSCN	*t*_(58)_ = − 2.71	**0.018**
		GJB2–NSCN	*t*_(50)_ = 0.64	0.656
Max slope	µV/nC	CND–GJB2	*t*_(42)_ = − 4.45	** < 0.001**
		CND–NSCN	*t*_(53)_ = − 3.18	**0.006**
		GJB2–NSCN	*t*_(56)_ = 1.94	0.104
	µV/dBC	CND–GJB2	*t*_(49)_ = − 3.27	**0.005**
		CND–NSCN	*t*_(69)_ = − 2.43	**0.033**
		GJB2–NSCN	*t*_(50)_ = 1.38	0.294
	dBV/dBC	CND–GJB2	*t*_(56)_ = − 3.17	**0.006**
		CND–NSCN	*t*_(67)_ = − 2.64	**0.020**
		GJB2–NSCN	*t*_(46)_ = 1.01	0.453
Linear slope	µV/nC	CND–GJB2	*t*_(42)_ = − 2.74	**0.018**
		CND–NSCN	*t*_(51)_ = − 3.68	**0.002**
		GJB2–NSCN	*t*_(53)_ = 0.49	0.732

**Table 4:** Post hoc tests for the effect of patient group at each electrode location on a subset of the interphase gap (IPG) effect measures, where the interaction between patient group and electrode location was statistically significant. *IPG* interphase gap, *CND* cochlear nerve deficiency, *GJB2* gap junction beta-2 mutation, *NSCN* normal-sized cochlear nerve, *nC* nanocoulomb, *µV* microvolt, *dBC* decibel relative to 1 nanocoulomb, *dBV* decibel relative to 1 microvolt, *dBC*_*linear/log*_ decibel relative to 1 nanocoulomb, calculated from the input/output (*I/O*) function quantified on a linear input/logarithmic output scale, *dBC*_*log/log*_ decibel relative to 1 nanocoulomb, calculated from the I/O function quantified on a logarithmic input/logarithmic output scale, *FDR* false discovery rate. Bolded text indicates *p*-values that are considered statistically significant (*p* < 0.05)

IPG effect measure	Quantitative scale	Electrode location	Patient group pair	Patient group effect	
				***t* value**	***p* value** (FDR adjusted)
Stimulation level offset	nC	3	CND–GJB2	*t*_(123)_ = 5.42	** < 0.001**
			CND–NSCN	*t*_(96)_ = 6.74	** < 0.001**
			GJB2–NSCN	*t*_(56)_ = 1.05	0.438
		12	CND–GJB2	*t*_(123)_ = 8.64	** < 0.001**
			CND–NSCN	*t*_(96)_ = 10.02	** < 0.001**
			GJB2–NSCN	*t*_(56)_ = 0.51	0.732
		21	CND–GJB2	*t*_(123)_ = 12.39	** < 0.001**
			CND–NSCN	*t*_(96)_ = 13.65	** < 0.001**
			GJB2–NSCN	*t*_(56)_ = − 0.44	0.754
	dBC_linear/log_	3	CND–GJB2	*t*_(91)_ = 0.07	0.961
			CND–NSCN	*t*_(147)_ = 1.36	0.294
			GJB2–NSCN	*t*_(65)_ = 0.92	0.470
		12	CND–GJB2	*t*_(91)_ = 3.09	**0.006**
			CND–NSCN	*t*_(147)_ = 5.87	** < 0.001**
			GJB2–NSCN	*t*_(65)_ = 0.90	0.471
		21	CND–GJB2	*t*_(91)_ = 3.68	**0.001**
			CND–NSCN	*t*_(147)_ = 5.54	** < 0.001**
			GJB2–NSCN	*t*_(65)_ = 0.01	0.990
	dBC_log/log_	3	CND–GJB2	*t*_(97)_ = − 0.20	0.903
			CND–NSCN	*t*_(147)_ = 0.99	0.453
			GJB2–NSCN	*t*_(68)_ = 1.00	0.453
		12	CND–GJB2	*t*_(97)_ = 2.76	**0.015**
			CND–NSCN	*t*_(147)_ = 5.39	** < 0.001**
			GJB2–NSCN	*t*_(68)_ = 1.11	0.421
		21	CND–GJB2	*t*_(97)_ = 3.36	**0.003**
			CND–NSCN	*t*_(147)_ = 5.08	** < 0.001**
			GJB2–NSCN	*t*_(68)_ = 0.20	0.903
Threshold	nC	3	CND–GJB2	*t*_(123)_ = − 4.01	** < 0.001**
			CND–NSCN	*t*_(106)_ = − 6.26	** < 0.001**
			GJB2–NSCN	*t*_(57)_ = − 1.10	0.421
		12	CND–GJB2	*t*_(123)_ = − 5.74	** < 0.001**
			CND–NSCN	*t*_(106)_ = − 7.57	** < 0.001**
			GJB2–NSCN	*t*_(57)_ = − 0.11	0.961
		21	CND–GJB2	*t*_(123)_ = − 10.47	** < 0.001**
			CND–NSCN	*t*_(106)_ = − 13.12	** < 0.001**
			GJB2–NSCN	*t*_(57)_ = 0.53	0.732
Max amplitude	dBV	3	CND–GJB2	*t*_(78)_ = − 0.93	0.470
			CND–NSCN	*t*_(144)_ = 0.34	0.823
			GJB2–NSCN	*t*_(68)_ = 1.18	0.395
		12	CND–GJB2	*t*_(78)_ = − 0.44	0.754
			CND–NSCN	*t*_(144)_ = 0.96	0.465
			GJB2–NSCN	*t*_(68)_ = 1.06	0.438
		21	CND–GJB2	*t*_(78)_ = 2.18	0.058
			CND–NSCN	*t*_(144)_ = 3.53	**0.002**
			GJB2–NSCN	*t*_(68)_ = − 0.07	0.961

**Table 5:** Post hoc tests for the effect of electrode location in each patient group on a subset of the interphase gap (IPG) effect measures, where the interaction between patient group and electrode location was statistically significant. *IPG* interphase gap, *CND* cochlear nerve deficiency, *GJB2* gap junction beta-2 mutation, *NSCN* normal-sized cochlear nerve, *nC* nanocoulomb, *µV* microvolt, *dBC* decibel relative to 1 nanocoulomb, *dBV* decibel relative to 1 microvolt, *dBC*_*linear/log*_ decibel relative to 1 nanocoulomb, calculated from the input/output (*I/O*) function quantified on a linear input/logarithmic output scale, *dBC*_*log/log*_ decibel relative to 1 nanocoulomb, calculated from the I/O function quantified on a logarithmic input/logarithmic output scale, *FDR* false discovery rate. Bolded text indicates *p*-values that are considered statistically significant (*p* < 0.05)

IPG effect measure	Quantitative scale	Patient group	Electrode location pair	Electrode location effect	
				***t* value**	***p* value** (FDR adjusted)
Stimulation level offset	nC	CND	3 − 12	*t*_(59)_ = − 2.87	**0.029**
			3 − 21	*t*_(59)_ = − 5.67	** < 0.001**
			12 − 21	*t*_(59)_ = − 2.80	**0.031**
		GJB2	3 − 12	*t*_(50)_ = 0.61	0.660
			3 − 21	*t*_(50)_ = 2.26	0.085
			12 − 21	*t*_(50)_ = 1.64	0.228
		NSCN	3 − 12	*t*_(76)_ = 0.24	0.866
			3 − 21	*t*_(76)_ = 2.05	0.113
			12 − 21	*t*_(76)_ = 1.80	0.169
	dBC_linear/log_	CND	3 − 12	*t*_(62)_ = − 0.66	0.652
			3 − 21	*t*_(62)_ = − 1.59	0.229
			12 − 21	*t*_(62)_ = − 0.93	0.503
		GJB2	3 − 12	*t*_(49)_ = 2.38	0.081
			3 − 21	*t*_(49)_ = 2.29	0.085
			12 − 21	*t*_(49)_ = − 0.08	0.934
		NSCN	3 − 12	*t*_(76)_ = 5.44	** < 0.001**
			3 − 21	*t*_(76)_ = 3.52	**0.005**
			12 − 21	*t*_(76)_ = − 1.92	0.139
	dBC_log/log_	CND	3 − 12	*t*_(65)_ = − 0.49	0.721
			3 − 21	*t*_(65)_ = − 1.53	0.241
			12 − 21	*t*_(65)_ = − 1.04	0.468
		GJB2	3 − 12	*t*_(49)_ = 2.45	0.073
			3 − 21	*t*_(49)_ = 2.26	0.085
			12 − 21	*t*_(49)_ = − 0.19	0.886
		NSCN	3 − 12	*t*_(74)_ = 5.53	** < 0.001**
			3 − 21	*t*_(74)_ = 3.45	**0.005**
			12 − 21	*t*_(74)_ = − 2.08	0.113
Threshold	nC	CND	3 − 12	*t*_(58)_ = 1.52	0.241
			3 − 21	*t*_(58)_ = 6.52	** < 0.001**
			12 − 21	*t*_(58)_ = 5.00	** < 0.001**
		GJB2	3 − 12	*t*_(48)_ = − 0.56	0.683
			3 − 21	*t*_(48)_ = − 1.21	0.386
			12 − 21	*t*_(48)_ = − 0.65	0.652
		NSCN	3 − 12	*t*_(81)_ = 0.98	0.481
			3 − 21	*t*_(81)_ = 0.81	0.557
			12 − 21	*t*_(81)_ = − 0.17	0.886
Max amplitude	dBV	CND	3 − 12	*t*_(74)_ = − 1.59	0.229
			3 − 21	*t*_(74)_ = − 3.63	**0.004**
			12 − 21	*t*_(74)_ = − 2.04	0.113
		GJB2	3 − 12	*t*_(44)_ = − 0.39	0.788
			3 − 21	*t*_(44)_ = 1.00	0.481
			12 − 21	*t*_(44)_ = 1.39	0.299
		NSCN	3 − 12	*t*_(75)_ = − 1.19	0.386
			3 − 21	*t*_(75)_ = − 0.30	0.840
			12 − 21	*t*_(75)_ = 0.89	0.517

**Fig. 7:**
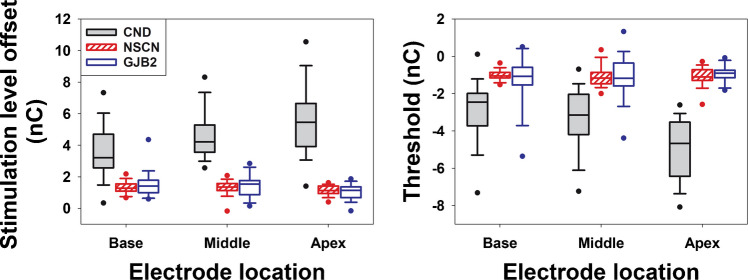
The interphase gap (IPG) effect quantified using the stimulation level (left panel) and the eCAP threshold (right panel) on a linear/linear scale in children with cochlear nerve deficiency (CND, filled boxes), children with normal − sized cochlear nerves (NSCNs, patterned boxes), and children with GJB2 mutations (open boxes). The line inside the box and box edges represents the median and the interquartile range (IQR), spanning the 25th to 75th percentiles, respectively. Whiskers indicate the most extreme data points within 1.5 times the IQR from the lower and upper quartiles. Dots falling outside the box indicate the 5th and 95th percentiles

Figure 7 illustrates the results of two variables that are sensitive to differences both among the three participant groups (pooled across electrode locations) and across electrode locations within the CND group. The left and right panels display stimulation level offset (in nC) and eCAP threshold (in nC) results, respectively. Data in both panels clearly show that children with CND exhibited a larger IPG effect, as indicated by greater deviations from zero for both parameters. Additionally, the magnitude of the deviation appeared to increase as the stimulation electrode location shifted toward more apical sites in children with CND. There is no apparent difference in either parameter between children with NSCN and those with GJB2 mutations.

The results of the LMMs confirmed these observations. Specifically, there was a significant main effect of participant group, as well as significant interactions between participant group and electrode location, on both the stimulation level offset (in nC) and the IPG effect on the eCAP threshold (in nC). Pairwise comparisons showed that children with CND exhibited significantly larger stimulation level offsets and greater IPG effects on the eCAP threshold than both children with NSCNs and those with GJB2 mutations. These group differences were consistently observed across all three electrode locations. No significant differences in either variable were found between the NSCN and GJB2 groups at any of the electrode locations tested. In children with CND, stimulation level offsets differed significantly between all pairs of electrode locations, with larger offsets observed at more apical sites. These participants also exhibited greater IPG effects on the eCAP threshold at the apical electrode location compared to the middle and the basal locations. No electrode-related effects on either variable were observed in the other two participant groups.

**Fig. 8:**
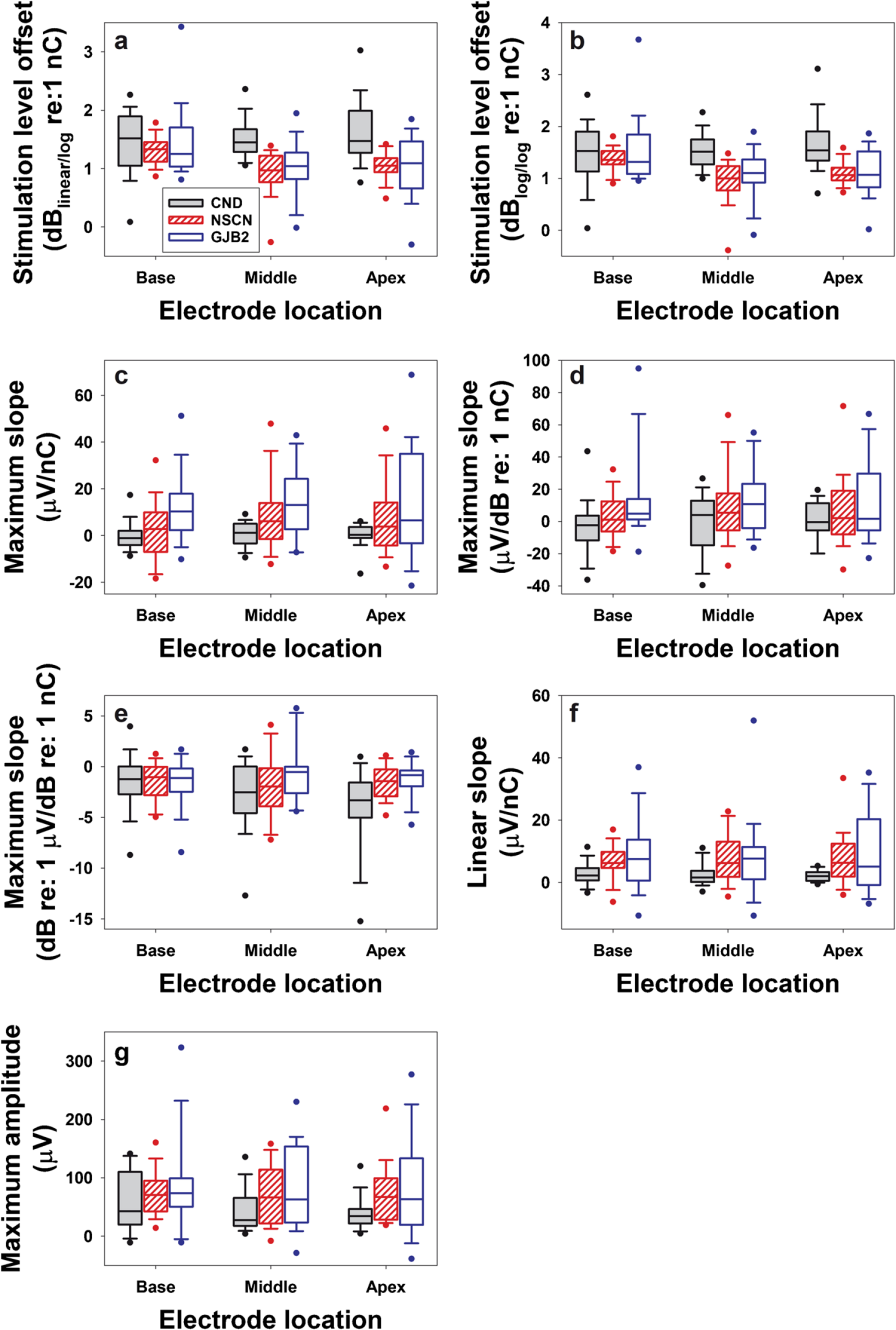
The interphase gap (IPG) effect quantified using the stimulation level offset (**a**,** b**), the maximum slope (**c**–**e**), the overall linear slope (**f**), and the maximum amplitude (**g**) on different scales in children with CND (filled boxes), children with NSCNs (patterned boxes), and children with GJB2 mutations (open boxes). The line inside the box and box edges represents the median and the interquartile range (IQR), spanning the 25th to 75th percentiles, respectively. Whiskers indicate the most extreme data points within 1.5 times the IQR from the lower and upper quartiles. Dots falling outside the box indicate the 5th and 95th percentiles

Figure 8 presents the results for seven variables that are sensitive to differences among the three participant groups but not across electrode locations within the CND group, with each panel representing one variable. Compared to the results shown in Fig. 7, these parameters exhibit much smaller variations across electrode locations in children with CND, despite clear differences among participant groups.

Aligned with these observations, LMMs showed significant main effects of participant group and electrode location, as well as significant interactions between the two factors on stimulation level offset in both dB_linear/log_ and dB_log/log_. For both variables, pairwise comparisons revealed that children with CND exhibited significantly larger stimulation level offsets than children with NSCNs and those with GJB2 mutations. These group differences were observed only at the middle and apical electrode locations. No significant differences in either variable were found between the NSCN and GJB2 groups at any electrode location. Children with CND showed no significant differences in the stimulation level offset between any two electrode locations calculated from eCAP I/O functions on either input/output scale. In contrast, children with NSCNs demonstrated significantly larger stimulation level offsets at the basal electrode location compared to both the middle and the apical electrode locations. Children with GJB2 mutations showed no significant across-electrode difference in stimulation level offsets calculated from eCAP I/O functions on either input/output scale.

Significant group differences were also found in the maximum slope quantified on linear/linear, linear/log, and log/log scales, the overall linear slope quantified on a linear/linear scale, and the maximum amplitude quantified on a linear scale (i.e., μV). For these variables, neither electrode location nor the interaction between participant group and electrode location reached statistical significance. Pairwise comparisons indicated that children with CND exhibited smaller IPG effects on these variables than both children with NSCNs and those with GJB2 mutations. No significant differences were found between the NSCN and GJB2 groups.

**Fig. 9:**
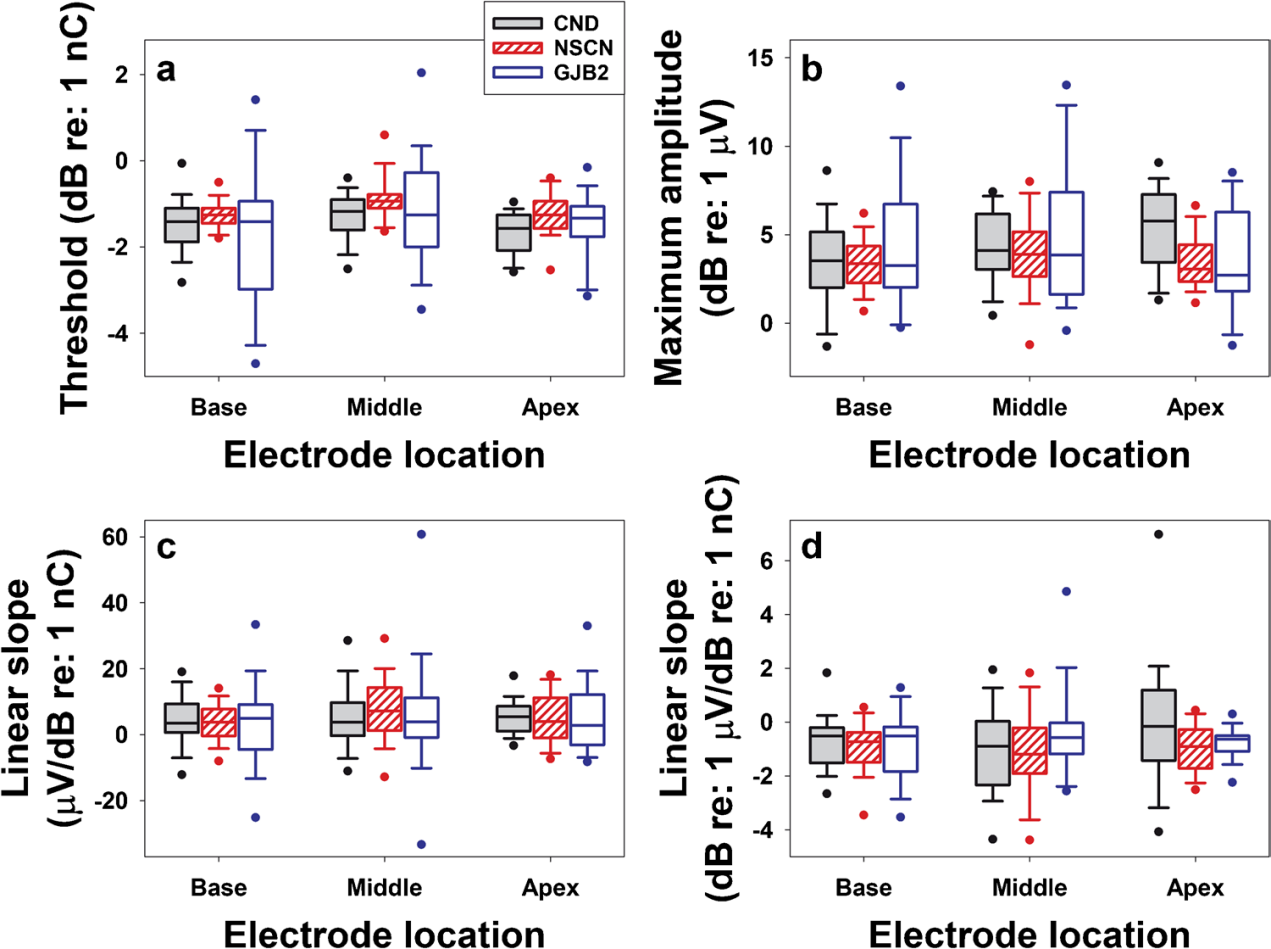
Interphase gap (IPG) effects on the eCAP threshold in dB (**a**), the maximum amplitude in dB, and the overall linear slope on linear/log (**c**) and log/log (**d**) scales in children with CND (filled boxes), children with NSCNs (patterned boxes), and children with GJB2 mutations (open boxes). The line inside the box and box edges represents the median and the interquartile range (IQR), spanning the 25th to 75th percentiles, respectively. Whiskers indicate the most extreme data points within 1.5 times the IQR from the lower and upper quartiles. Dots falling outside the box indicate the 5th and 95th percentiles

Figure 9 depicts the results for four variables that are not sensitive to differences in the number of CN fibers among participant groups or across electrode locations in children with CND, with each panel representing one variable. The maximum amplitude (in dB) and the overall linear slope measured on linear/log and log/log scales do not demonstrate any evidence for potential effects of group or electrode. Consistent with this observation, the LMMs revealed no significant main effects of participant group, electrode location, or their interaction on the overall linear slope measured on linear/log and log/log scales. For the maximum amplitude (in dB), the LMM revealed no significant main effects of participant group or electrode location, but a significant interaction between the two factors. Pairwise comparisons indicated that children with CND exhibited a larger IPG effect on the maximum amplitude at the apical electrode compared to the basal electrode. No significant differences were observed between other electrode pairs in children with CND, nor across electrode locations in children with NSCNs or those with GJB2 mutations. Children with NSCNs appeared to show a smaller IPG effect on eCAP threshold (in dB) than the other two participant groups across all three electrode locations. Consistent with these observations, the LMM revealed significant main effects of participant group and electrode location, but no significant interaction between the two factors. Post hoc analyses indicated that children with CND had a significantly larger IPG effect on eCAP threshold than children with NSCNs, whereas there were no significant differences between children with CND and GJB2 mutations or between children with GJB2 mutations and NSCNs. The absence of a group difference in IPG effect on eCAP threshold (in dB) between children with CND and GJB2 mutations—two populations that differ in the number of relatively healthy CN fibers—suggests that this measure is unlikely to be a reliable indicator of the number of activated CN fibers.

## Discussion

This study aimed to (1) determine whether the IPG effect primarily reflects the number or the health of available CN fibers and (2) identify the most informative and scientifically robust parameters, analytical methods, and quantitative scales for evaluating the IPG effect in human cochlear implant (CI) users. To address these objectives, IPG effects were measured in three pediatric patient groups expected to differ in anatomical characteristics of CN fibers, as suggested by histological results from human temporal bone studies, knowledge of inner ear embryogenesis, and the functional role of connexin 26. The findings for each aim are discussed in the following sections.

### Biological Basis of the IPG Effect

For this aim, we hypothesized that the IPG effect primarily reflects the number of available CN fibers responsive to electrical stimulation. To test this hypothesis, neural synchrony in the CN, a property dependent on the health of activated CN fibers, and the IPG effect were compared between children with CND and children with GJB2 mutations. In addition, the associations between the PLV—an index for quantifying neural synchrony in the CN—and the IPG effect assessed using different parameters, analytical methods, and quantitative scales were evaluated in children with GJB2 mutations.

Our results revealed no significant difference in the PLV between children with CND and children with GJB2 mutations. Given the well-established evidence linking neural synchrony to neural health [12, 50–54], this finding suggests comparable neural health of the CN between these two patient populations. These results are consistent with histological data from human temporal bone studies, which show relatively healthy CN fibers in both patient populations, despite substantial differences in the number of existing CN fibers [35, 36, 46]. In contrast, significant group differences were observed in all but four variables used to evaluate the IPG effect between children with CND and those with GJB2 mutations (Figs. 7, 8, and 9). The exceptions are likely influenced by non-neural factors, which are discussed in the following section. Furthermore, the absence of significant associations between the PLV and the IPG effect across all tested variables in children with GJB2 mutations suggests that these two measures reflect distinct biological processes. Taken together, these findings support our hypothesis that the IPG effect is more strongly influenced by the number of electrically responsive CN fibers than by their health. This interpretation is further supported by previous work by Ramekers et al. [20, 22], Takanen et al. [25], and Zhang et al. [27], reinforcing the view that the IPG effect may serve as a proxy for estimating the quantity, rather than the quality, of surviving/existing CN fibers.

The absence of a significant difference in the IPG effect between children with NSCNs and those with GJB2 mutations warrants caution in interpreting the IPG effect as a direct index of the number of activated CN fibers. The estimated total number of CN fibers in a typical cochlea is approximately 35,500 [34]. Taking into account the age-related loss of roughly 100 neurons per year [34] and the mean age at testing for children with GJB2 mutations in this study (approximately 11 years) and assuming no additional CN fiber loss due to cochlear implantation, the estimated CN fiber count in this group is around 34,400. In contrast, the average number of CN fibers in children with NSCNs is reported to be approximately 15,500 [47]. Despite this substantial difference—nearly 19,000 fibers—the lack of an observable difference in the IPG effect between these two groups suggests a limited sensitivity of the IPG effect to large variations in the number of electrically responsive CN fibers. However, this interpretation is complicated by the significant group difference observed between children with CND and those with NSCNs, who differ by approximately 10,000 fibers.

This apparent inconsistency is unlikely to be attributable to the shorter pulse phase duration (25 μs/phase) used to measure the IPG effect in six ears from five children with GJB2 mutations, compared with that used in other participants, because shorter pulse phase durations are generally associated with larger IPG effects [20, 65]. Instead, this inconsistency may be explained by a possible nonlinear relationship between the IPG effect and CN fiber count. Specifically, we speculate that the IPG effect is more responsive to changes in neural population size when the number of surviving fibers is already markedly reduced. In such cases, additional losses may have a more pronounced functional impact, which is captured by the IPG effect. Conversely, when CN fiber counts are reasonably good, even substantial differences may not significantly affect the IPG measurement. This speculated nonlinear association may account for the lack of difference observed between the GJB2 and NSCN groups, despite their large numerical disparity in CN fiber counts. This speculation cannot be directly tested in human CI users due to the inability to measure the number of electrically responsive CN fibers with sufficient precision. Therefore, studies using animal models or computational modeling approaches are warranted to provide mechanistic insights into the relationship between CN fiber count and the IPG effect.

It is important to note that the simulation study reported by Takanen et al. [25] was based on a relatively idealized modeling framework, incorporating up to 2000 healthy CN fibers across the entire cochlea and excluding many factors known to influence eCAP recordings, such as stimulation and recording artifacts, variability in elicited current, and recording noise. Under these idealized conditions, a linear relationship between the number of activated CN fibers and the IPG effect on maximum slope (quantified using a linear/linear scale; see their Fig. 9a) was observed. It remains an open question to what extent simulation results obtained from this relatively idealized computational model generalize to more realistic recording environments that include additional, potentially uncharacterized sources of variability. Addressing this question and determining the linear versus non-linear relationship between CN fiber count and the IPG effect requires coordinated, complementary efforts from CI manufacturers and researchers with diverse expertise. These efforts include, but are not limited to, improving eCAP measurement systems and artifact-reduction paradigms to minimize electrical artifact contamination and device-related noise in experimental data, as well as refining computational CN models based on human CI data to explore a broader range of CN degenerative conditions in simulations.

It is important to emphasize that our findings do not suggest that the IPG effect lacks clinical relevance or that it cannot serve as a useful indicator of CI outcomes. At present, there is no definitive evidence identifying the minimum number of CN fibers required to support good CI performance across different listening environments. Therefore, further research is warranted to clarify the clinical implications of the IPG effect, better define its role in predicting CI outcomes, and evaluate its potential as a clinical tool for optimizing programming parameters.

### Parameters, Analytical Methods, and Quantitative Scales

To identify the most informative and scientifically robust parameters, analytical methods, and quantitative scales for evaluating the IPG effect, the IPG effect measured at three electrode locations across the electrode array was quantified using different parameters, analytical methods, and quantitative scales and compared among children with CND, children with GJB2 mutations, and children with NSCNs. Our results demonstrated that both stimulation level offset and the eCAP threshold, when analyzed on linear scales, were sensitive to differences in CN fiber count across participant groups and electrode locations within the CND group. Additionally, we identified seven variables—including the stimulation level offset calculated from eCAP I/O functions on both linear/log and log/log scales, the maximum amplitude on a linear scale, the maximum slope on all scales, and the overall linear slope on a linear/linear scale—that were sensitive only to group differences. In contrast, IPG effects on the maximum amplitude on a logarithmic scale and on the overall linear slope on both linear/log and log/log scales were not sensitive to differences in CN fiber count across participant groups or across electrode locations within the CND group. Finally, although the LMM revealed a significant difference in IPG effect on eCAP threshold (in dB) between children with CND and NSCNs, the absence of a group difference between children with CND and GJB2 mutations indicates that this variable is not a reliable indicator of CN fiber count.

#### Parameters

Among all parameters examined in this study, differences in the stimulation level offset and the IPG effect on the maximum slope were consistently observed between children with CND and the other two pediatric participant groups, regardless of the quantitative scales applied. When calculated from eCAP I/O functions on a linear/linear scale (in nC), the stimulation level offset in children with CND also corresponded to the expected variation in CN fiber count across different intracochlear locations. These findings support the use of the stimulation level offset and the IPG effect on the maximum slope as reliable indicators of CN fiber counts in CI users. In contrast, the degree to which IPG effects on the maximum amplitude, the overall linear slope, and the eCAP threshold corresponded to expected group differences based on CN fiber count varied with the quantitative scale, indicating that these parameters lack robustness as group-level indicators.

Even though the IPG effect on the eCAP threshold (in nC) captured differences in CN fiber count both among patient populations and across electrode locations within children with CND, we advise careful consideration before using it as a reliable parameter to use for assessing the IPG effect due to three reasons. First, the determination of the eCAP threshold is impacted by the level of recording noise for eCAP measurements, which varies substantially across manufacturers. For example, the noise floor has been reported to be approximately 20 μV in older Cochlear™ Nucleus® implants and 2–5 μV in newer models [60]. In Advanced Bionics devices, the reported noise floor ranges from 20 to 50 μV [66]. For MED-EL devices, the noise floor was reported to be frequency dependent, with an estimated overall level of 2.6–10 µV [67–69]. It should be noted that the methods used to estimate the noise floor differed across studies. To date, no direct comparison of noise floors across devices using the same methodology has been reported, complicating the interpretation of how noise levels may impact eCAP threshold determination—whether based on experimenter expertise or computational algorithms [69]—across different manufacturers. This hardware-related limitation cannot be addressed through alternative analytical or quantitative methods and poses a significant challenge when comparing results across patients with different devices. Second, the determination of the presence or absence of the eCAP response at near threshold level based on visual inspection during eCAP recording depends on the experimenter’s experience and criteria. Less experienced or more conservative experimenters may require stronger responses to confirm the eCAP, and consequently, may stop recording at higher stimulation levels compared to more experienced ones. This introduces experimenter bias into the study results, further compromising the accuracy of threshold determination, which in turn affects the reliability of the IPG effect on the eCAP threshold. Finally, a significant correlation between the IPG effect on the eCAP threshold and CN fiber counts has not been consistently observed across studies (e.g., [20, 70]), and the relationship between these measures appears to be modulated by other eCAP stimulation parameters, such as pulse phase duration [20]. These results further question the validity of the IPG effect on the eCAP threshold as a robust and reliable indicator of CN fiber count.

#### Analytical Methods and Quantitative Scales

The results shown in Figs. 3 and 4 suggest that applying different input and output scales alters the overall shape of some I/O functions, while others remain largely unaffected. This occurs due to the compressive scaling introduced by the logarithmic transformation. Specifically, applying a logarithmic transformation to the output of a linear function results in an exponential shape due to the compressive nature of the logarithmic function at high values and its steep gradient at low values. This nonlinear scaling distorts the original linear relationship, emphasizing differences at lower values and compressing those at higher values.

**Fig. 10:**
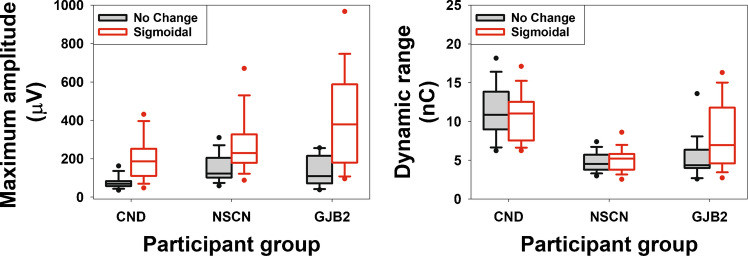
Maximum amplitudes and dynamic ranges were measured for eCAP input/output functions that either followed the same shape (black) or transitioned to an exponential shape (red) when the display scale was changed to a log/log format, across different participant groups. The line inside the box and box edges represents the median and the interquartile range (IQR), spanning the 25th to 75th percentiles, respectively. Whiskers indicate the most extreme data points within 1.5 times the IQR from the lower and upper quartiles. Dots falling outside the box indicate the 5th and 95th percentiles

We counted the number of cases in which the eCAP I/O functions transitioned to an exponential shape when the display scale was changed to a log/log format, based on visual inspection in each of three participant groups. Among children with CND, there were 16 cases (10.8%) and 25 cases (26.9%) at IPGs of 7 μs and 42 μs, respectively. In children with NSCNs, the number of cases increased to 59 (63.4%) for both IPGs. Among children with GJB2 mutations, 35 cases were observed, making up 61.8% of the group. Mann-Whitney *U* tests were used to compare maximum amplitudes and dynamic ranges between electrode locations where the eCAP I/O functions followed a similar shape versus those transitioned to an exponential shape when the display scale was changed to a log/log format (example electrodes shown in Fig. 4). The dynamic range was defined as the difference in stimulation level between the eCAP threshold and the C level. Significance levels of these *U* tests were adjusted using the Bonferroni correction. Consistent with the mathematical explanation provided above, Mann-Whitney *U* tests revealed that cases exhibiting a transition to an exponential shape had significantly larger maximum eCAP amplitudes compared to those that remained unchanged. In contrast, no significant differences in dynamic range were observed, except in the 7 μs IPG condition for children with GJB2 mutations. These results suggest that eCAP amplitude is the major factor determining the shape of I/O function on different scales. Full statistical details are provided in Supplemental Digital Content 2. Figure 10 presents the results for the 42 μs IPG condition across the three participant groups as an illustrative example, which closely resemble those obtained at 7 μs. MCLs for each participant group are shown in Supplemental Digital Content 3 to demonstrate that the fewer cases in which eCAP I/O functions transitioned to an exponential shape on a log/log scale in children with CND were not due to lower MCLs in these participants.

This variability influences the estimation of both the maximum slope (via the window method) and the overall linear slope (via linear regression). Specifically, due to the compressive nature of the logarithmic transformation, an eCAP I/O function that follows an exponential distribution exhibits a steeper slope at lower input levels than at higher input levels. Consequently, when using the window method, the estimated maximum slope primarily reflects the rate of amplitude growth near threshold levels. This differs from the maximum slope estimates obtained using linear regression in cases where the I/O function appears more linear. As a result, this inflates data variability due to the analytical method rather than reflecting true differences in the underlying physiological data. However, in our dataset, we did not observe a significant impact of this issue on the results or the overall conclusions regarding the IPG effect on the maximum slope (Fig. 8), despite the group differences showing an opposite trend compared to the results obtained using both linear/linear and linear/log scales.

A sigmoidal regression function was used to quantify stimulation level offset. As reported by He et al. [23] using the same dataset, this function provides a reasonably good fit to the eCAP I/O functions when expressed on a linear/linear scale, as evidenced by mean goodness-of-fit values of 0.89 and 0.90 for children with CND and children with NSCNs, respectively. As illustrated in Figs. 3 and 4, the overall shape of the eCAP I/O function is largely preserved when the input axis is transformed from a linear to a logarithmic scale, as dynamic range does not primarily determine its shape. Consequently, the goodness-of-fit of the sigmoidal model is not expected to change substantially with this transformation. However, when expressed on a log/log scale, eCAP I/O functions from some participants exhibited an exponential profile. To ensure scientific rigor, we therefore compared the goodness of fit of the sigmoidal regression function with that of an exponential model of the form

$$y(x)={\mathrm{y}}_{0}+a(1-{e}^{-bx})$$

where $y(x)$ represents the eCAP amplitude, ${\mathrm{y}}_{0}$ denotes the eCAP amplitude measured at the eCAP threshold level, *a* is the range of eCAP amplitude, and *b* is the slope, with larger values indicating steeper functions.

**Fig. 11:**
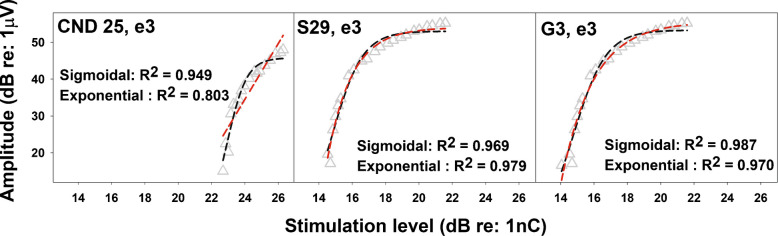
Fitting results of sigmoidal (black dashed lines) and exponential (red dashed lines) regression models for a subset of eCAP input/output (I/O) functions shown in Fig. 4. These eCAP I/O functions were measured at an interphase gap of 42 µs and are displayed on a log/log scale. Participant number, electrode location, and adjusted *R*^2^ values indicating goodness of fit for both models are shown in each panel

Our results showed that although the exponential function provided a better fit in some cases, it performed worse in others relative to the sigmoidal regression. As an illustrative example, Fig. 11 presents the fitting results of both models for a subset of the eCAP I/O functions shown in Fig. 4, measured with an IPG of 42 µs and displayed on a log/log scale. Overall, our findings did not indicate that the exponential model provided a systematically better fit than the sigmoidal regression for the eCAP functions included in this study, supporting the use of sigmoidal regression to calculate the stimulation level offset.

**Fig. 12:**
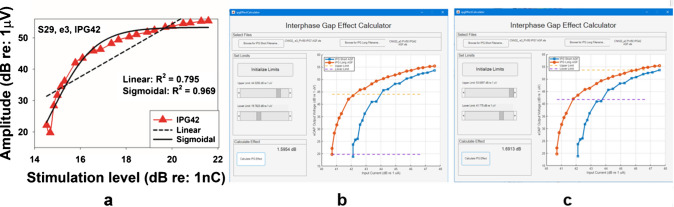
An illustration of the issue arising from the use of linear regression to estimate the slope of an eCAP input/output (*I/O*) function that follows an exponential distribution and to calculate the stimulation level offset. **a** The eCAP I/O function measured at electrode 3 with an interphase gap (IPG) of 42 µs in a child with a normal-sized cochlear nerve (participant S29). The results of linear and sigmoidal regressions are indicated by dashed and solid lines, respectively. The goodness of fit (adjusted *R*^2^) for each model is displayed within the panel. **b, c** The output from the tool described by Brochier et al. [26]. In each panel, symbols connected by blue and red lines represent I/O functions measured at IPGs of 7 µs and 42 µs, respectively. The region between the two horizontal dashed lines indicates the selected segment used for stimulation level offset calculation

It is apparent that linear regression is not expected to yield accurate estimates when the data exhibit an exponential distribution. As illustrated in panel (a) of Fig. 12, linear regression (dashed line) fails to capture the characteristics of the example eCAP I/O function and results in a poorer fit compared to a sigmoidal function (solid line) [44]. Excluding specific data points from the I/O function to force a linear fit is not recommended, as it not only distorts the experimental data but also increases analytical error. This limitation also affects the quantification of stimulation level offsets based on linear regression results, such as the method proposed by Brochier et al. [25]. To better illustrate the issue, we calculated stimulation level offset from eCAP I/O functions on a log/log scale for this example using the MATLAB tool provided by Brochier et al. [25], available at https://github.com/tjbrochier/eCAP-AGF-Methods. Panels (b) and (c) show screenshots of the tool’s output, demonstrating that two different portions of the I/O function could potentially be selected for calculating the stimulation level offset. While both portions appear relatively linear, they correspond to different regions of the I/O function. The segment shown in panel (b) lies in the lower part of the function. The stimulation level offsets calculated based on the segments shown in panels (b) and (c) are 1.59 dB and 1.69 dB, respectively.

The use of logarithmic scales for both input and output to display the eCAP I/O function is recommended by Brochier et al. [26] based on formula-derivation results of a theoretical model expressed as:

$$V=rn (sg{I)}^{2}$$

where *r* represents the characteristics of the recording electrode, *n* denotes the number of the neurons near the stimulating electrode, *s* reflects the characteristics of the stimulating electrode, *g* is a stimulus-dependent gain factor, and *I* indicates the input electrical current level. After a logarithmic transformation, all factors in the model become additive. When calculating the stimulation level offset, the effects of *r*, *n*, and *s* are eliminated, as these represent shared, constant factors across both IPG conditions. Based on these calculations, Brochier et al. concluded that stimulation level offset computed on a log/log scale is not influenced by non-neural, electrode-related factors and primarily reflects the health of the activated CN fibers. For detailed explanations, please refer to Brochier et al. [26]. The mathematical derivation itself is valid. However, eCAPs represent a spatially weighted summation of CN fiber activity that is affected by both neural and non-neural factors (for a review, see Miller et al. [71]). In addition to CN fibers located near the stimulating electrode, CN fibers closer to the recording electrode also play a significant role in generating eCAP responses [71], a factor that is not considered in the theoretical model proposed by Brochier et al. [26]. Under conditions of cross-turn stimulation [72], in which a basal electrode activates CN fibers originating from more apical cochlear turns, and in cases of widespread neural excitation [73], CN fibers located farther from both the stimulating and recording electrodes may also contribute to the measured eCAP. These complexities are likewise not incorporated into the theoretical model of Brochier et al. [26]. These issues may account for the discrepancy in the proposed biological basis of the IPG effect between our study and that reported by Brochier et al. [26]. In addition, our results shown in Figs. 7 and 8 do not indicate that using a log/log scale yields better outcomes for detecting group differences in CN counts, either for the stimulation level offset or for the IPG effect on the maximum slope. In fact, only the stimulation level offset calculated from eCAP I/O function on a linear/linear scale was able to reflect differences in CN fiber count across electrode locations in children with CND. In this case, the “effective” distance between the electrode and the target CN fibers increases at more apical locations, not due to changes in physical electrode placement, but rather as a result of the reduced CN fiber count. When a log/log scale is applied, the effect of this fiber reduction is effectively eliminated. While a logarithmic scale for quantifying input stimulation level can reduce the influence of physical electrode-to-modiolus distance caused by electrode placement [74], it also masks the “physiological” electrode-to-modiolus distance resulting from CN fiber loss near the target electrode. This is a relevant concern in CI users, given the well-documented loss of CN fibers in this population [e.g., [1, 3, 4, 40, 75]. At this point, it remains unclear whether physical or physiological distance is the more influential factor affecting the IPG effect. Given the similarity in results across different scaling methods for both parameters, we do not recommend one particular scale over the others for comparing across patient groups. These findings suggest that the robustness of these parameters as indicators of CN fiber counts is preserved across commonly used scaling approaches. However, depending on the research question, the stimulation level offset calculated from eCAP I/O function on a linear/linear scale may be preferable.

### Study Limitations

This study has five limitations. First, and most importantly, the distinct anatomical characteristics of CN fibers in the three pediatric patient populations were inferred from histological findings from human temporal bone studies, established knowledge of inner ear embryogenesis, and the functional role of connexin 26. Because histological data from patients with CND and GJB2 mutations are scarce, and direct assessment of CN histology in living human participants is not feasible, it remains unknown whether the existing CN fibers in children with CND and those with GJB2 mutations are as healthy as those in normal-hearing listeners. Second, postoperative CT scans are not routinely performed as part of clinical care for pediatric CI users, which limits our ability to evaluate the potential influence of electrode-to-modiolus distance, insertion angle, or insertion depth on the IPG effect. To reduce variability associated with these electrode-placement factors, this study included only CI users implanted with a perimodiolar electrode array when comparing the IPG effect. This practice, together with evidence from animal models and human CI users showing a nonsignificant impact of electrode impedance and electrode-to-modiolus distance on the IPG effect [30, 76–78], helps reduce the potential influence of these factors on our results. Nevertheless, the potential effects of insertion angle and insertion depth on the IPG effect were not evaluated and warrant further studies. Third, adult CI users were not included in this study due to the substantial variability in CN fiber counts reported in the literature. Specifically, mean CN fiber counts reported in adults with severe-to-profound hearing loss or CI users range from 7,510 [75], a value comparable to that observed in children with CND, to 18,533 [2], a number similar to counts reported for children with NSCNs. Including adult CI users would therefore introduce additional variability and complicate the interpretation of study results. Fourth, the absence of differences in neural health between study populations was assessed here using the PLV method. Other metrics, such as the polarity effect on eCAP threshold, have also been proposed to evaluate neural health (e.g., [29, 79]). However, this metric could not be applied in the present study, as all eCAPs were measured using only cathodic-leading, charge-balanced biphasic pulses. Finally, sex- and gender- based analyses were not conducted in this study, which may be considered a limitation.

## Conclusions

The IPG effect primarily reflects CN fiber counts rather than fiber health. Regardless of the quantitative scale applied, the stimulation level offset estimated using sigmoidal regression and the difference in the maximum slope measured at different IPGs can be used as reliable indicators of CN fiber counts in CI users.

## Supplementary Information

Below is the link to the electronic supplementary material.

Supplementary file1 (DOCX 22.5 KB)

Supplementary file2 (DOCX 15.3 KB)

Supplementary file3 (DOCX 151 KB)

## Data Availability

The data that support the findings of this study are available from the authors upon reasonable request with permission from The Ohio State University.

## Declarations

### Disclaimer

No AI technology was used to write content, generate data, or create figures.
