# Supplementary material for: The Interphase Gap Effect in Cochlear Implant Users: Biological Basis, Parameter Selection, Analytical Methods, and Quantitative Scales
Source: J Assoc Res Otolaryngol. 2026 Mar 16;27(3):465–90. doi: 10.1007/s10162-026-01041-3 (PMC13237389; doi:10.1007/s10162-026-01041-3)
Supplement: Supplementary file 1 — Supplementary file1 (DOCX 22.5 KB) [file 10162_2026_1041_MOESM1_ESM.docx]

**Table 1A**. Demographic information of subjects who participated in He et al. (2020). L, left; R, right; CND, cochlear nerve deficiency; CI24RE (CA), Freedom Contour Advance electrode array; PM: peri-modiolar.

| Participant number | Ear tested | Age at testing (yrs) | Internal device and electrode array | Electrode array type | Electrode tested |
| --- | --- | --- | --- | --- | --- |
| CND1 | L | 8.4 | 24RE (CA) | PM | 1,3,7 |
| CND2 | R | 6.9 | 24RE (CA) | PM | 3,12,21 |
| CND3 | L | 4.1 | 24RE (CA) | PM | 1,3,6 |
| CND4 | L | 2.5 | 24RE (CA) | PM | 1,5,10 |
| CND5 | R | 3.4 | 24RE (CA) | PM | 3,12,21 |
| CND6 | L | 23.1 | 24RE (CA) | PM | 3,9,16 |
| CND7 | L | 6.1 | 24RE (CA) | PM | 1,4,7 |
| CND8 | R | 8.5 | 24RE (CA) | PM | 1,4,8 |
| CND9 | R | 1.9 | 24RE (CA) | PM | 1,4,8 |
| CND10 | L | 2.4 | 24RE (CA) | PM | 3,12,21 |
| CND11 | R | 4.1 | 24RE (CA) | PM | 1,5,8 |
| CND12 | R | 6.5 | 24RE (CA) | PM | 3,12,21 |
| CND13 | L | 3.3 | 24RE (CA) | PM | 3,12,21 |
| CND14 | L | 5.4 | 24RE (CA) | PM | 3,10,18 |
| CND15 | R | 4.1 | 24RE (CA) | PM | 3,12,21 |
| CND16 | L | 2.4 | 24RE (CA) | PM | 1,7,14 |
| CND17 | L | 12.1 | 24RE (CA) | PM | 3,10,17 |
| CND18 | L | 7.8 | 24RE (CA) | PM | 1,3,7 |
| CND19 | R | 7.8 | 24RE (CA) | PM | 3,12,20 |
| CND20 | L | 9.1 | 24RE (CA) | PM | 3,12,21 |
| CND21 | L | 2.5 | 24RE (CA) | PM | 3,12,21 |
| CND22 | L | 8.0 | 24RE (CA) | PM | 3,12,21 |
| CND23 | R | 8.5 | 24RE (CA) | PM | 3,6,10 |
| CND24 | L | 5.2 | 24RE (CA) | PM | 3,12,21 |
| CND25 | R | 2.4 | 24RE (CA) | PM | 1,2,3 |
| CND26 | L | 6.5 | 24RE (CA) | PM | 3,12,21 |
| CND27 | L | 4.3 | 24RE (CA) | PM | 1,5,11 |
| CND28 | L | 7.3 | 24RE (CA) | PM | 2,10,19 |
| CND29 | L | 3.3 | 24RE (CA) | PM | 1,3,4 |
| CND30 | R | 3.5 | 24RE (CA) | PM | 1,6,11 |
| S1 | R | 4.4 | 24RE (CA) | PM | 3,12,21 |
| S2 | R | 2.6 | 24RE (CA) | PM | 3,12,21 |
| S3 | L | 3.1 | 24RE (CA) | PM | 3,12,21 |
| S4 | R | 3.6 | 24RE (CA) | PM | 3,12,21 |
| S5 | R | 7.9 | 24RE (CA) | PM | 3,12,21 |
| S6 | R | 2.1 | 24RE (CA) | PM | 3,12,21 |
| S7 | R | 8.3 | 24RE (CA) | PM | 3,12,21 |
| S8 | R | 4.8 | 24RE (CA) | PM | 3,12,21 |
| S9 | R | 5.0 | 24RE (CA) | PM | 3,12,21 |
| S10 | R | 6.7 | 24RE (CA) | PM | 3,12,21 |
| S11 | R | 7.2 | 24RE (CA) | PM | 3,12,21 |
| S12 | R | 7.0 | 24RE (CA) | PM | 3,12,21 |
| S13 | L | 9.6 | 24RE (CA) | PM | 3,12,21 |
| S14 | L | 4.0 | 24RE (CA) | PM | 3,12,21 |
| S15 | L | 4.2 | 24RE (CA) | PM | 3,12,21 |
| S16 | R | 9.7 | 24RE (CA) | PM | 3,12,21 |
| S17 | R | 5.1 | 24RE (CA) | PM | 3,12,21 |
| S18 | R | 2.7 | 24RE (CA) | PM | 3,12,21 |
| S19 | R | 13.1 | 24RE (CA) | PM | 3,12,21 |
| S20 | R | 2.9 | 24RE (CA) | PM | 3,12,21 |
| S21 | L | 2.1 | 24RE (CA) | PM | 3,12,21 |
| S22 | L | 3.3 | 24RE (CA) | PM | 3,12,21 |
| S23 | R | 10.8 | 24RE (CA) | PM | 3,12,21 |
| S24 | L | 3.5 | 24RE (CA) | PM | 3,12,21 |
| S25 | L | 4.6 | 24RE (CA) | PM | 3,12,21 |
| S26 | R | 9.6 | 24RE (CA) | PM | 3,12,21 |
| S27 | L | 10.3 | 24RE (CA) | PM | 3,12,21 |
| S28 | L | 4.0 | 24RE (CA) | PM | 3,12,21 |
| S29 | R | 4.9 | 24RE (CA) | PM | 3,12,21 |
| S30 | R | 7.4 | 24RE (CA) | PM | 3,12,21 |
