# Supplementary material for: The Interphase Gap Effect in Cochlear Implant Users: Biological Basis, Parameter Selection, Analytical Methods, and Quantitative Scales
Source: J Assoc Res Otolaryngol. 2026 Mar 16;27(3):465–90. doi: 10.1007/s10162-026-01041-3 (PMC13237389; doi:10.1007/s10162-026-01041-3)
Supplement: Supplementary file 2 — Supplementary file2 (DOCX 15.3 KB) [file 10162_2026_1041_MOESM2_ESM.docx]

**Table 2A**. Mann-Whitney U test results comparing maximum amplitude and dynamic range of eCAP input/output functions that either maintained a linear form or transitioned to a sigmoidal form upon changing the display scale to log-log format, across three participant groups. CND, cochlear nerve deficiency; NSCN, normal sized cochlear nerve; GJB2, Gap Junction Beta-2.

| Participant Group | Interphase Gap (μs) | Maximum Amplitude (μV) | Dynamic Range (nC) |
| --- | --- | --- | --- |
| CND | 7 | U = 5.24, *p* < .001 | U = 0.98, *p* = .328 |
|  | 42 | U = 5.50, *p* < .001 | U = -1.01, *p* = .313 |
| NSCN | 7 | U = 5.77, *p* < .001 | U = 1.99, *p* = .047 |
|  | 42 | U = 4.36, *p* < .001 | U = 1.12, *p* = .262 |
| GJB2 | 7 | U = 4.33, *p* < .001 | U = 2.88, *p* = .004 |
|  | 42 | U = 4.44, *p* < .001 | U = 2.52, *p* = .012 |
