# Supplementary material for: The Interphase Gap Effect in Cochlear Implant Users: Biological Basis, Parameter Selection, Analytical Methods, and Quantitative Scales
Source: J Assoc Res Otolaryngol. 2026 Mar 16;27(3):465–90. doi: 10.1007/s10162-026-01041-3 (PMC13237389; doi:10.1007/s10162-026-01041-3)
Supplement: Supplementary file 3 — Supplementary file3 (DOCX 151 KB) [file 10162_2026_1041_MOESM3_ESM.docx]

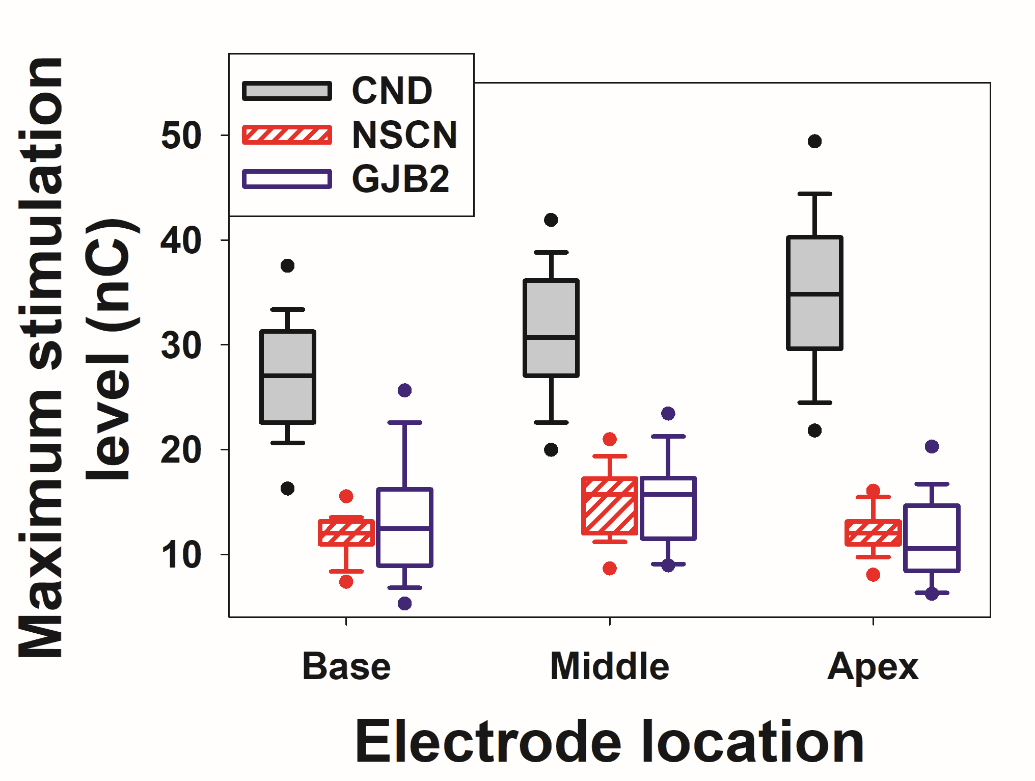


**Figure 1A**. Maximum stimulation levels (MCLs) for measuring the electrically evoked compound action potential at three electrode location in children with cochlear nerve deficiency (CND, filled boxes), children with normal-sized cochlear nerves (NSCNs, patterned boxes), and children with GJB2 mutations (open boxes). The line inside the box and box edges represents the median and the interquartile range (IQR), spanning the 25th to 75th percentiles, respectively. Whiskers indicate the most extreme data points within 1.5 times the IQR from the lower and upper quartiles. Dots falling outside the box indicate the 5th and 95th percentiles. These data indicate substantially higher MCLs in children with CND compared with the other participant groups.
